# Full-visibility 3D imaging of oxygenation and blood flow by simultaneous multispectral photoacoustic fluctuation imaging (MS-PAFI) and ultrasound Doppler

**DOI:** 10.1038/s41598-023-29177-9

**Published:** 2023-02-20

**Authors:** Guillaume Godefroy, Bastien Arnal, Emmanuel Bossy

**Affiliations:** Univ. Grenoble Alpes, LIPhy, CNRS, Grenoble, 38000 France

**Keywords:** Optical imaging, Ultrasound, Imaging and sensing

## Abstract

We present a method and setup that provide complementary three-dimensional (3D) images of blood oxygenation (via quantitative photoacoustic imaging) and blood flow dynamics (via ultrasound Doppler). The proposed approach is label-free and exploits blood-induced fluctuations, and is implemented on a sparse array with only 256 elements, driven with a commercially available ultrasound electronics. We first implement 3D photoacoustic fluctuation imaging (PAFI) to image chicken embryo, and obtain full-visibility images of the vascular morphology. We obtain simultaneously 3D ultrasound power Doppler with a comparable image quality. We then introduce multispectral photoacoustic fluctuation imaging (MS-PAFI), and demonstrate that it can provide quantitative measurements of the absorbed optical energy density with full visibility and enhanced contrast, as compared to conventional delay-and-sum multispectral photoacoustic imaging. We finally showcase the synergy and complementarity between MS-PAFI, which provides 3D quantitative oxygenation (SO$_2$) imaging, and 3D ultrasound Doppler, which provides quantitative information on blood flow dynamics. MS-PAFI represents a promising alternative to model-based inversions with the advantage of resolving all the visibility artefacts without prior and regularization, by use of a straightforward processing scheme.

## Introduction

Vascular imaging is an essential tool in medicine, and is provided by a number of imaging modalities, including state-of the art clinical techniques such as ultrasound imaging^1–3^, magnetic resonance imaging^4,5^ (MRI) and X-rays computerized tomography^6^ (CT), or more recent techniques such as photoacoustic imaging^7–9^. In this work, we focus on the synergistic combination of ultrasound Doppler and photoacoustic imaging for three-dimensional (3D) vascular imaging, without contrast agents. Ultrasound Doppler techniques have been used intensely in clinical practice for decades in various disciplines^1^, and are still undergoing major conceptual and technological advances for vascular imaging such as ultrafast Doppler^2^ and functional ultrasound imaging^3^. Ultrasound Doppler techniques indeed provide a broad range of information such as vasculature morphology, blood content, flow direction and quantification of blood flow speed and pulsatility. However, ultrasound cannot probe molecular information, such as the blood oxygenation, which can only be accessed through complementary techniques such as diffuse optical tomography (DOT)^10^, blood-oxygen-level-dependent (BOLD) magnetic resonance imaging (MRI)^5^ or photoacoustic imaging^7–9^. Photoacoustic/optoacoustic imaging is an emerging noninvasive imaging modality that provides optical absorption contrasts with the depth and resolution of ultrasound imaging^8^, at least within the light penetration depth. It is particularly suited for vascular imaging thanks to the strong optical absorption of hemoglobin^11^. In addition, photoacoustic spectroscopy (involving multi-wavelength acquisitions) has the ability to provide quantitative maps of chromophore concentrations and blood oxygenation^9,12,13^. Compared to other non invasive techniques that are sensitive to blood oxygenation such as DOT^10^ and BOLD-MRI^5^, the advantages of vascular photoacoustic imaging include its large depth-to-resolution ratio^13^ (on the order of 200) and its relatively low cost. Moreover, because ultrasound imaging and photoacoustic imaging both rely on detecting ultrasound signals, with similar acquisition hardware and similar image reconstruction principles, they have a huge potential to be combined synergistically to provide a comprehensive characterization of vascular networks, including vascular morphology, blood flow dynamics and blood oxygenation.

The combination of ultrasound and photoacoustic imaging has been the topic of earlier investigations, but with significant limitations as compared to the approach proposed here. Most of the investigations currently reported in the literature are restricted to conventional pulse-echo ultrasound imaging coupled with photoacoustic imaging^14–19^, whereas state-of-the-art label-free Doppler techniques, such as implemented in our work, provide access to functional ultrasound imaging and have never been coupled to photoacoustic imaging. In addition, many of these investigations are restricted to 2D imaging^14–18^, while most structures including vascular networks are inherently three-dimensional. Three-dimensional vascular imaging in general has been the topic of intensive research for both ultrasound imaging and photoacoustic imaging^20–25^, in each specific community. Strategies for 3D imaging are commonly based on the assumption that reconstructing full-visibility images (unaffected by structural corruption) involves full-view detection with a Nyquist-based spatial sampling, leading to approaches that require mechanical scanning and/or fully populated matrix arrays (typically ~ 1024 elements^21^). Another issue arises for photoacoustic imaging, as the limited-view and the limited bandwidth situations lead to the well known visibility artefacts^26–28^. In the context of vascular imaging, such visibility artefacts consist of partially or totally invisible vessels. Model-based reconstructions^29^ can effectively restore a good image quality in the limited view/bandwidth case and when the number of elements remains limited (typically ~ 512). However, there are drawbacks in using such approaches: (1) to compensate for the limited-bandwidth of the probe, the signals are usually deconvolved by the impulse response of the probe which may increase the effect of low frequency noise. (2) Model-based reconstruction algorithms have very high computational costs. (3) Applying model-based approaches on experimental data often requires prior knowledge and subtle tuning of regularization parameters, which increases complexity and may limit its practical use. Overall, model-based reconstructions, especially in 3D, require very specific developments and expertise. (5) Finally, while these approaches can partially compensate for limited-bandwidth artefacts, they can not recover all the structures in the limited view scenario^30^.

To date, only one publication reported both 3D ultrasound and 3D photoacoustic imaging with the same system, based on a custom 512-element spherical array^30^. However, ultrasound images were obtained by injecting microbubbles as ultrasound contrast agent, and the system remained limited in terms of aperture detection and limited view effects were observed in the mouse brain. Currently, none of the proposed system for 3D photoacoustic imaging can provide full-visibility images without mechanical scanning or contrast agents.

To circumvent the limited-view problem in photoacoustic imaging, we recently introduced and experimentally demonstrated photoacoustic fluctuation imaging^31^ (PAFI), as a powerful method that provides full-visibility photoacoustic images of the blood vasculature, even under limited-view conditions. In particular, we obtained preliminary label-free full-visibility 3D photoacoustic images in a chicken embryo, at a single optical wavelength. However, single-wavelength PAFI provides purely morphological images of vascular networks, thus with little or no complementary information as compared to that provided by ultrasound Power Doppler (US-PwD). To fully exploit the potential of photoacoustic imaging, multispectral acquisition is required to access quantitative molecular information such as blood oxygenation^9,12^. Multispectral 3D photoacoustic imaging was demonstrated in early investigations^23,28,32–34^, but either required mechanical scanning or suffered from visibility artefacts when implemented with a fixed array^30^. In this work, we extend the PAFI approach by introducing multispectral photoacoustic fluctuation imaging (MS-PAFI), and show that it can provide 3D, full-visibility and quantitative maps of blood oxygenation (SO$_2$ values), even with a sparse array and under limited-view conditions. To do so, we extend the theoretical framework of PAFI, from which we introduce multispectral photoacoustic fluctuation imaging (MS-PAFI). Additionnaly, we couple MS-PAFI to 3D ultrasound Doppler imaging for further blood flow characterization. For both modalities, 3D images are obtained with a static hemispherical transducer probe with only 256 elements, driven by a standard commercially available electronics and using standard delay-and-sum DAS algorithm for image reconstructions. MS-PAFI could allow any user and platform to enhance photoacoustic imaging performance at a low implementation cost. The absence of deconvolution, prior knowledge (besides speed-of-sound of the medium) or regularization ensures straightforward quantitative measurements. Finally, the capabilities of our approach are showcased *in vivo* through 3D Doppler and 3D blood oxygenation (SO$_2$) images obtained simultaneously on chicken embryos.

## Results

### Experimental setup

**Figure 1:**
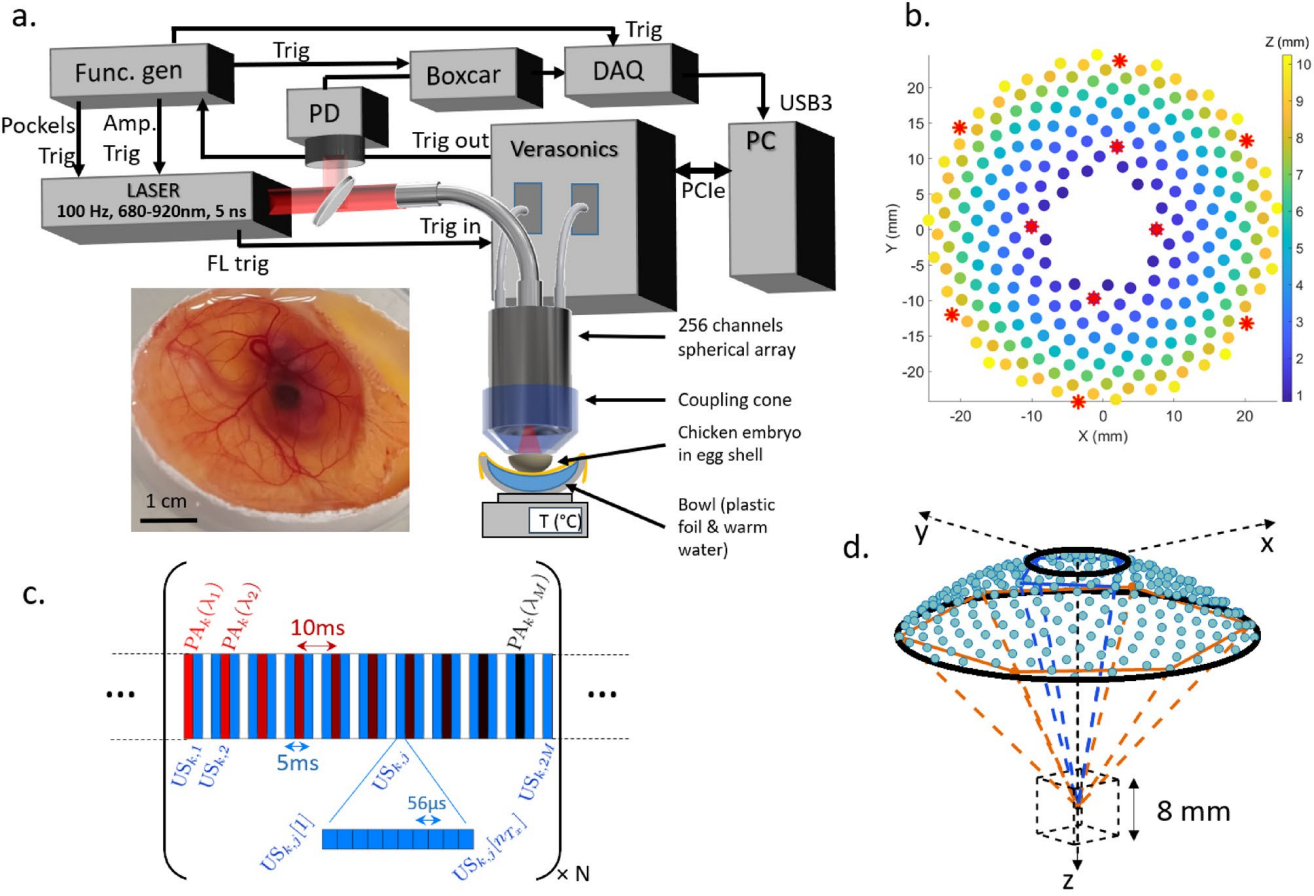
Experimental setup for simultaneous photoacoustic and ultrasound 3D imaging. (**a**) Schematic overview of the setup (see detailed description in the “Materials and methods” section). *PD* photodiode, *DAQ* data acquisition card. *PC* computer. (**b**) Sunflower distribution of the 256 elements projected in the XY plane; color-coding indicates depth (z direction). (**c**) A complete aquisition resulted from the repeated acquisition of N sequences. Each sequence (indexed with subscript $k=1...N$) consisted in the formation of *M* multispectral 3D photoacoustic images (100 Hz imaging rate) and $2 \times M$ 3D ultrasound image (200 Hz imaging rate). Each 3D ultrasound image resulted from the ultrafast acquisition (17.8 kHz frame rate) of $n_{T_x}=10$ emissions by each of the $n_{T_x}$ elements depicted as red stars on (**d**). (**d**) 3D rendering of the elements positions and the volumetric imaging field-of-view.

The experimental setup and measurement scheme allowing for simultaneous ultrasound and photoacoustic 3D imaging is represented schematically in Fig. 1a. It is based on a custom 256-element spherical matrix array (center frequency 8 MHz), connected to a commercially available 256-channel ultrasound electronics. With only 256 elements distributed over a spherical surface for 3D imaging (Fig. 1b), the probe is sparse, in the sense that it has much fewer elements than traditionally required by the Nyquist spatial sampling rate. Its elements are distributed over a non-periodic sunflower pattern (Fig. 1b) in order to reduce grating lobes induced by the spatial sub-sampling^35^, and their directivity results in a volumetric field-of-view of approximately $8 \times 8 \times 8\, \text {mm}^3$ (Fig. 1d). For multispectral photoacoustic imaging, a tunable OPO laser is used to deliver nanosecond light pulses to the sample via a fiber bundle passing through the center of the matrix array. The optical wavelength can be set for each individual laser pulse, at a fixed repetition rate of 100 Hz, and a photodiode can record the energy of each pulse. The ultrasound electronics is capable of both emitting and receiving ultrasound signals, with a repetition rate up to several kHz, and thus allows implementing both state-of-the-art ultrasound imaging sequences (including ultrafast ultrasound imaging and ultrasound Doppler techniques) and photoacoustic imaging.

For the results presented in this work, we used N repeated sequences of interleaved ultrasound and photoacoustic acquisitions, as represented schematically in Fig. 1c (each individual sequence is indexed with subscript $k=1...N$). For each sequence, $M=10$ sets of photoacoustic radiofrequency (RF) data, corresponding to *M* wavelengths, were acquired at 100 Hz (laser repetition rate) in an interleaved manner with $2 \times M$ sets of ultrasound RF data (see details in the “Materials and methods” section). A complete acquisition finally resulted in a set of $N\times M$ multispectral 3D photoacoustic images (100 Hz imaging rate) and $2\times N\times M$ 3D ultrasound image (200 Hz imaging rate). Each individual 3D ultrasound image resulted from the ultrafast acquisition (17.8 kHz) of $n_{Tx}=10$ ultrasound measurements. All results from live chicken embryos presented in the manuscript were obtained with the shell partially removed, as illustrated in Fig. 1a.

### Simultaneous ultrasound power Doppler and photoacoustic fluctuation imaging

We first illustrate the unique capability of the proposed setup to provide simultaneously *both* 3D photoacoustic fluctuation images (PAFI) and 3D ultrasound Power Doppler images (US-PwD), in addition to conventional DAS photoacoustic images and conventional ultrafast ultrasound images. Both PAFI and US-PwD rely on selectively extracting the temporal fluctuation of a series of single-shot delay-and-sum (DAS) 3D images. From a full acquisition of N repeated sequences, *M* photoacoustic fluctuation images are obtained from the N DAS 3D photoacoustic images acquired for each wavelength, and one 3D Power Doppler image is obtained from up to $2\times M \times N$ ultrafast ultrasound images. The various data-processing steps for PAFI^31^ and US-PwD^36^ are strictly identical, and we will refer to fluctuation images for both modalities. Briefly, a singular-value-decomposition (SVD) filtering approach is used to selectively extract the fluctuations induced by flowing red blood cells from all other parasitic sources of fluctuations, following the approach proposed in earlier works^31,36^. For both PAFI and US-PwD, two parasitic sources of fluctuations are electronics noise and tissue motion. Additional sources of fluctuations in PAFI include the laser pulse energy fluctuations^31^, and the choice of the SVD-filtering parameters thus depends on each modality. All data-processing steps are detailed in the “Materials and methods” section.

**Figure 2:**
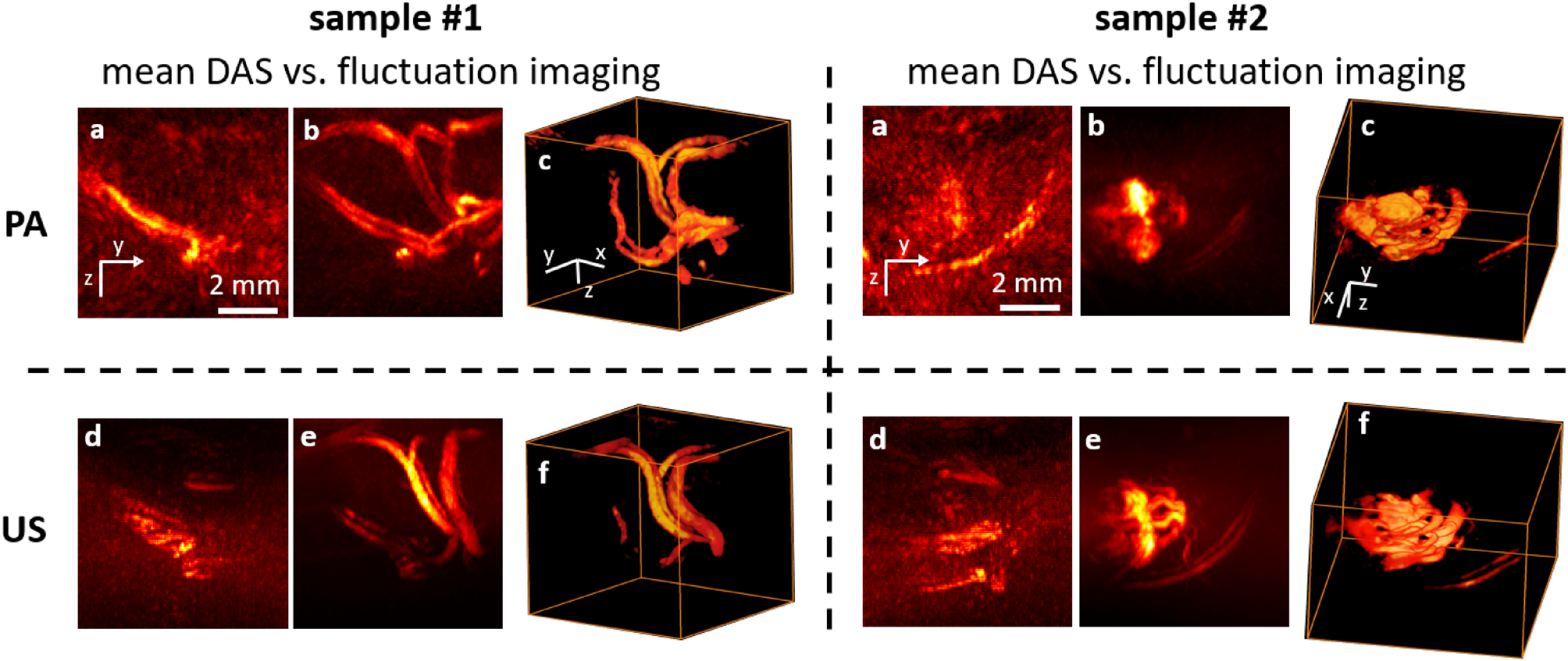
Three-dimensional photoacoustic fluctuation imaging (PAFI, (**b,c**), top row) and Ultrasound Power Doppler (US-PwD, (**e,f**), bottom row) imaging, along with DAS photoacoustic imaging (**a**) and DAS ultrafast ultrasound imaging (**d**). The images represent blood vasculature obtained in two different representative chicken embryos. Photoacoustic images correspond to photoacoustic signals acquired at $\lambda =720\ \text{nm}$. (**a,b,d,e**) maximum intensity projections (MIP) along the x-direction through 3D images. (**c,f**) 3D rendering.

Figure 2 shows representative results of PAFI and US-PwD obtained on two different chicken embryos. In this work, for the sake of consistency and comparison, we define fluctuation images as standard-deviation images for both PAFI and US-PwD. For US-PwD, variance images may also be found in the literature, as directly related to the so-called “blood volume” parameter^37^. For each modality, the conventional DAS images, (a) and (d), computed from the average of *N* photoacoustic images and $2\times M\times N$ ultrasound images, suffer from both visibility artefacts and a strong background. The origin of the visibility artefacts was described in detail in earlier works^26,27,31^: structures oriented along the z-axis, emitting waves mostly outside the detection aperture are absent from the reconstructed images. The strong background is a direct consequence of the probe sparsity: while the non-periodic spatial distribution of the transducer elements prevents from grating lobes, the unfocused energy due to spatial sub-sampling remains present as a significant pseudo-random background^35^. As a major consequence, our proposed setup has a relatively low contrast to background ratio for conventional DAS imaging. However, when fluctuation imaging is considered (b, c, e and f), the visibility artefacts are eliminated *and* the parasitic background is significantly reduced: vessels oriented along the z-axis are clearly revealed in fluctuation images, whereas they are invisible in conventional DAS images. In addition, as expected from previous results^31,38,39^, the resolution of fluctuation images is increased as compared to conventional DAS images, with pair of close vessels clearly resolved only on fluctuations images. As a key consequence, fluctuation imaging enables 3D label-free imaging of blood vessels with a sparse matrix array and a commercially available 256-channel ultrasound electronics, which we demonstrate here simultaneously for PAFI and US-PwD.

### Principles of multispectral photoacoustic fluctuation imaging (MS-PAFI)

As PAFI provides similar qualitative morphological information on blood vessels as compared to US-PwD, its major added-value resides in its potential to provide quantitative molecular information such as blood oxygenation, a physiological parameter inaccessible to ultrasound Doppler techniques. In this part, we demonstrate both theoretically and experimentally that multispectral PAFI allows to measure specifically the spectral dependence of the optical energy absorbed by blood, $\mu _\text{RBC}(\lambda ,{\mathbf{r}})\, \Phi (\lambda ,{\mathbf{r}})$, where $\mu _\text{RBC}(\lambda ,{\mathbf{r}})$ is the absorption coefficient within red blood cells (RBC) and $\Phi (\lambda ,{\mathbf{r}})$ is the local fluence at position ${\mathbf{r}}$ and wavelength $\lambda$. In photoacoustic fluctuation imaging, the relevant physical parameters to describe the statistical properties of RBCs are^31^ their volume fraction $\eta ({\mathbf{r}})$, the volume $V_\text{RBC}({\mathbf{r}})$ of a single RBC, the so-called packing factor $W(\eta )$ that reflects correlations between neighboring RBC^31,40^, and a binary function $f_\text{vessels}({\mathbf{r}})$ that describes the geometry of the vascular network ($f_\text{vessels}({\mathbf{r}})=1$ inside blood vessels, $f_\text{vessels}({\mathbf{r}})=0$ outside). We note $h_{\text{PA}}({\mathbf{r}})$ the complex-valued 3D point spread function (PSF) of the photoacoustic imaging modality, which entirely characterize the imaging system, including the laser, the ultrasound transducer and the ultrasound electronics^31^. Under the assumption that both the light fluence and all the physical parameters of blood are constant at the scale of the PSF, but may vary from one vessel to another, the theoretical expressions for the mean photoacoustic image ($m_\text{PA}=E[\text{PA}_k](\lambda ,{\mathbf{r}})$) and the photoacoustic fluctuation image of blood vessels ($\displaystyle \sigma _\text{PA}=\sigma [\text{PA}_k]=\sqrt{E [\text{PA}_{k}^2]-E[\text{PA}_k]^2}$) provided in our previous work^31^ can be readily adapted to take into account the wavelength and spatial dependence of all relevant physical properties:

1 $$\begin{aligned} m_\text{PA}(\lambda ,{\mathbf{r}})= & {} \Gamma ({\mathbf{r}})\, \mu _\text{RBC}(\lambda ,{\mathbf{r}})\, \Phi (\lambda ,{\mathbf{r}})\, \eta ({\mathbf{r}})\, \left| f_\text{vessels}\times h_{\text{PA}}\right| ({\mathbf{r}}), \end{aligned}$$

2 $$\begin{aligned} \sigma _\text{PA}(\lambda ,{\mathbf{r}})= & {} \Gamma ({\mathbf{r}})\, \mu _\text{RBC}(\lambda ,{\mathbf{r}})\, \Phi (\lambda ,{\mathbf{r}})\, \sqrt{\eta ({\mathbf{r}})\, W[\eta ({\mathbf{r}})]\, V_\text{RBC}({\mathbf{r}})\, \left[ f_\text{vessels}\times |h_{\text{PA}}|^2\right] ({\mathbf{r}})}. \end{aligned}$$

In experiments, the mean and fluctuation images are estimated from the *N* images available at each wavelength. These expressions clearly show that the mean image and the fluctuation image have the exact same dependence on $\Gamma ({\mathbf{r}}) \, \mu _\text{RBC}(\lambda ,{\mathbf{r}}) \, \Phi (\lambda ,{\mathbf{r}})$, which demonstrates that multispectral PAFI carries the same quantitative optical information as conventional photoacoustic imaging. Exactly as for conventional photoacoustic imaging, multispectral PAFI can thus be expected in theory to provide access to the wavelength dependence of $\mu _\text{RBC}(\lambda ,{\mathbf{r}}) \, \Phi (\lambda ,{\mathbf{r}})$ at each pixel of the image, although with a different local proportionality coefficient $\displaystyle K_{\sigma _{\text{PA,blood}}}({\mathbf{r}})=\Gamma ({\mathbf{r}})\,\sqrt{\eta ({\mathbf{r}}) \, W[\eta ({\mathbf{r}})] \, V_\text{RBC}({\mathbf{r}})\, \left[ f_\text{vessels}\times |h_{\text{PA}}|^2\right] ({\mathbf{r}})}$. A major difference between the two type of images is related to their dependence on the properties of RBCs (concentration and volume), on the structure of the blood vessels described by $f_\text{vessels}({\mathbf{r}})$, and very importantly on the type of convolution involved in the image formation, $\left| f_\text{vessels}\times h_{\text{PA}}\right|$ for conventional photoacoustic imaging as opposed to $\left[ f_\text{vessels}\times |h_{\text{PA}}|^2\right]$) for PAFI. As previously investigated in detail in our earlier work^31^, the major consequence is that PAFI provides images of the vasculature free of visibility artefacts even for limited-view detection, thanks to the $\left[ f_\text{vessels}\times |h_{\text{PA}}|^2\right]$ dependence, where conventional imaging only provides visibility-limited images (as illustrated by Fig. 2). As another major difference, whereas conventional multispectral photoacoustic imaging requires specific spectral unmixing technique to discriminate the absorption of blood from that of other chromophores^9,12^, PAFI automatically unmixes the contribution of flowing RBCs from all other static chromophores, and is therefore intrinsically *specific* to blood.

As the first theoretical result of our work, multispectral PAFI can thus in principle provide the same quantitative information as conventional imaging, but with enhanced resolution and with full visibility. However, Eq. (2) above considers the fluctuation induced by flowing RBCs only. As mentioned in the previous section, photoacoustic images may also fluctuate via several other parasitic sources, including measurement noise from the ultrasound electronics, electromagnetic radiation from the laser power supply, laser pulse energy fluctuation, temporal laser jitter and tissue motion. In the following, we extend our theory of PAFI to include the additional fluctuations induced by the laser pulse energy fluctuations from shot to shot and by the electronic noise. While the laser fluctuations could in principle be compensated by recording the energy of each pulse, we will show that this is not necessary, greatly simplifying the practical implementation of multispectral PAFI. The local fluence for each $k{\text {th}}$ laser shot is noted as $\Phi _{k}(\lambda ,{\mathbf{r}})$, and can be conveniently formulated as $\Phi _{k}(\lambda ,{\mathbf{r}}) = {\overline{\Phi }}(\lambda ,{\mathbf{r}})[1+\delta _{k}(\lambda )]$, where ${\overline{\Phi }}(\lambda ,{\mathbf{r}})$ is the mean local fluence and $\delta _{k}(\lambda )$ the *relative* laser pulse energy fluctuation. By definition $<\delta _{k}(\lambda )>=0$, and we note $\sigma ^2_{\text{laser}}(\lambda )=\left<\delta _{k}^2(\lambda )\right>$. The influence of the electronic noise on RF data is modelled as a random stationary background noise on single-shot photoacoustic images, which corresponds to a homogeneous fluctuation background on fluctuation image, noted as $\sigma _n$. Under the assumption that the three sources of fluctuations are independent, the total *variance* fluctuation image may be written as the sum of three terms (see demonstration in Supplementary Information):

3 $$\begin{aligned} \sigma ^2_\text{PA}(\lambda ,{\mathbf{r}}) = \bigg [1+\sigma _{\text{laser}}^2(\lambda )\bigg ]\, \sigma ^2_{\text{PA,blood}}(\lambda ,{\mathbf{r}})+ \sigma ^2_{\text{laser}}(\lambda )\, m_\text{PA}^2(\lambda ,{\mathbf{r}}) + \sigma _n^2, \end{aligned}$$

where $\displaystyle \sigma _\text{PA,blood}(\lambda ,{\mathbf{r}}) =\mu _\text{RBC}(\lambda ,{\mathbf{r}}) \, {\overline{\Phi }}(\lambda ,{\mathbf{r}}) \, K_{\sigma _{\text{PA,blood}}}({\mathbf{r}})$ is the ideal blood-induced-only fluctuation image, and the mean image $m_\text{PA}(\lambda ,{\mathbf{r}})=\Gamma ({\mathbf{r}}) \, \mu _\text{RBC}(\lambda ,{\mathbf{r}}) \, {\overline{\Phi }}(\lambda ,{\mathbf{r}}) \, \eta ({\mathbf{r}})\, \left| f_\text{vessels}\times h_{\text{PA}}\right| ({\mathbf{r}})$ is the mean image with the average fluence value. Equation (3) constitutes the first major theoretical result of our work, and indicates that both the electronic noise $\sigma _n$ and the laser pulse energy fluctuation $\sigma ^2_{\text{laser}}(\lambda )$ must be taken into account in PAFI in order to retrieve quantitative information proportional to $\mu _\text{RBC}(\lambda ,{\mathbf{r}}) \, {\overline{\Phi }}(\lambda ,{\mathbf{r}})$. If the pulse energy fluctuation is too large, the second term of Eq. (3) may become dominant, in which case the fluctuation image $\sigma _{\text{PA}}(\lambda ,{\mathbf{r}})$ simply becomes proportional to the visibility-limited mean image $m_\text{PA}(\lambda ,{\mathbf{r}})$, in agreement with experimental results reported in our previous work^31^. The relative contribution of each term depends on the specific experimental conditions: for the results presented in Fig. 2, the second term $\sigma ^2_{\text{laser}}(\lambda )\, m^2(\lambda ,{\mathbf{r}})$ was typically 5 to 10 times higher than the first term of interest, and the background fluctuation term $\sigma _n^2$ was approximately only two times smaller than the first term of interest. Removing the two parasitic fluctuation terms therefore turns out to be absolutely necessary to retrieve $\sigma _{\text{PA,blood}}(\lambda ,{\mathbf{r}})$, moreover in a quantitative manner. To do so, following the approach introduced in our earlier work^31^, a SVD filtering processing step was applied to the full stack of $N\times M$ photoacoustic images $\text{PA}_{k}(\lambda ,{\mathbf{r}})$: removing singular vectors with the highest singular values allows discarding the influence of the pulse energy fluctuations (second term in Eq. (3)). For our laser, $\sigma ^2_{\text{laser}}(\lambda )$ was of the order of $0.2\ \%$, and its influence on the first fluctuation term could thus be neglected ($[1+\sigma _{\text{laser}}^2(\lambda )]\simeq 1.00$).

We note $\text{PA}_{k,\text{SVD}}$ the corresponding SVD-filtered images. It is assumed that the level of background noise is unaffected by the SVD processing step, as the noise is distributed over all singular vectors, from which only a small fraction (typically a few percent) are filtered out by the SVD. The computation of the variance image for each wavelength provides a stack of *M* SVD-filtered variance images, given by $\displaystyle \sigma ^2_\text{PA,SVD}(\lambda ,{\mathbf{r}}) = \sigma ^2_{\text{PA,blood}}(\lambda ,{\mathbf{r}}) + \sigma _{n}^2$. We finally obtain an estimation for the fluctuation induced by blood-only via the following expression:

4 $$\begin{aligned} \sigma _{\text{PA,blood}}(\lambda ,{\mathbf{r}})=\mu _\text{RBC}(\lambda ,{\mathbf{r}}) \, {\overline{\Phi }}(\lambda ,{\mathbf{r}}) \, K_{\sigma _{\text{PA,blood}}}({\mathbf{r}})=\sqrt{\sigma ^2_\text{PA,SVD}(\lambda ,{\mathbf{r}}) - \sigma ^2_{n}}. \end{aligned}$$

Equation (4) constitutes the second major theoretical result of our work: it indicates that upon proper SVD filtering and background noise subtraction, multispectral PAFI provides access to the relative spectral dependence of $\mu _\text{RBC}(\lambda ,{\mathbf{r}}) \, \Phi (\lambda ,{\mathbf{r}})$ at each pixel of the reconstructed volume. Multispectral PAFI thus provides the exact same quantitative information as conventional multispectral imaging, but gives this information even for structures that would otherwise be invisible on conventional imaging, as the objective and benefit of PAFI. Importantly, we note that, as for conventional multi-spectral imaging, it still remains necessary for MS-PAFI to know the relative spectral dependence of the local fluence $\Phi (\lambda ,{\mathbf{r}})$ in order to derive the local relative spectral dependence of the absorption coefficient $\mu _\text{RBC}(\lambda ,{\mathbf{r}})$, and finally derive SO$_2$ values. In the following, we provide experimental demonstrations of MS-PAFI in proof-of-concept situations were the fluence is known, i.e. in the absence of scattering. When the relative fluence spectrum is known, Eq. (4) demonstrates that it is hence possible to measure the relative spectral dependence of the absorption coefficient of blood at each pixel of the photoacoustic image, and derive further quantitative measurements such as the blood oxygen saturation (see “Materials and methods” section).

**Figure 3:**
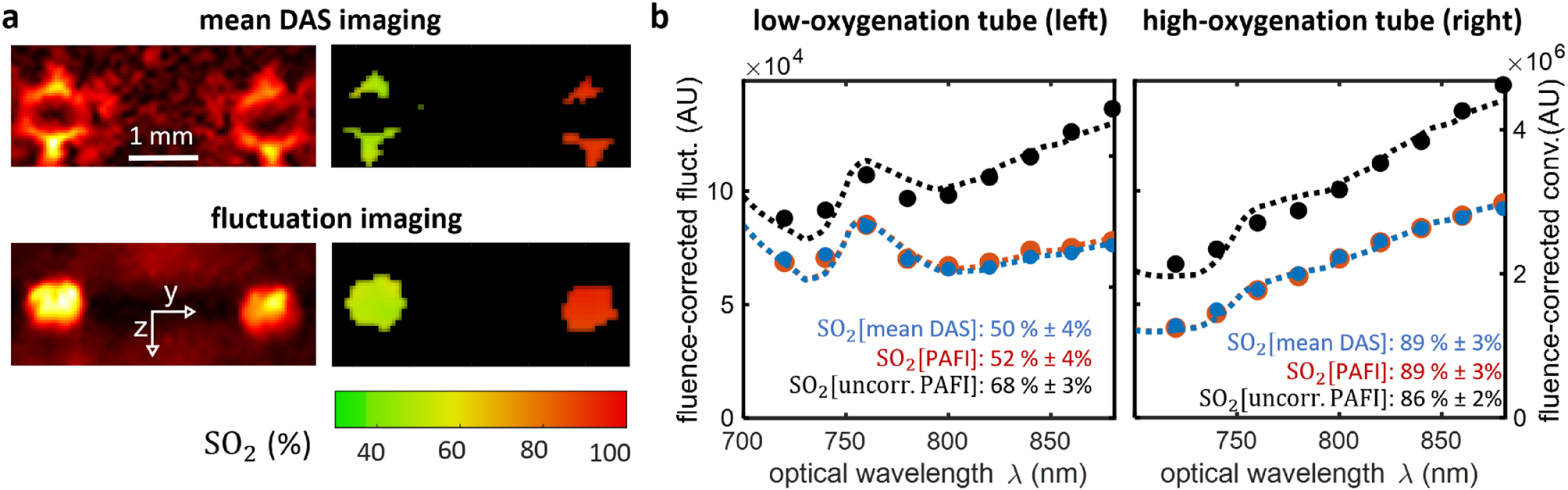
In vitro validation of multispectral photoacoustic fluctuation imaging (MS-PAFI) through a non-scattering medium. (**a**) 2D cross-sectional photoacoustic images of transparent capillary glass tubes (10 $\upmu \text {m}$ wall thickness) filled with flowing blood at two different oxygenation levels. Top row: DAS photoacoustic imaging. Bottom row: MS-PAFI. MS-PAFI provides the same quantitative SO$_2$ values as conventional DAS photoacoustic imaging, but without visibility artefacts. (**b**) Relative spectral dependence of $\mu _\text{RBC}(\lambda ,{\mathbf{r}})$ estimated over the regions of interest (ROIs) indicated in (**a**): blue, red and black estimates are respectively for conventional DAS PA imaging, PAFI and uncorrected PAFI (without noise subtraction). The dashed lines represent the model curve that best fits the MS-PAFI data, leading to the fit-estimated SO$_2$ value. ± values for SO$_2$ are the spatial standard deviation computed over the ROIs (the ROIs correspond to regions were SO$_2$ values were computed).

### In vitro validation of MS-PAFI

We first experimentally validated the above principles in vitro in a non-scattering medium by measuring the spectral dependence of $\mu _\text{RBC}(\lambda ,{\mathbf{r}})$ and subsequent SO$_2$ levels in two transparent capillary glass tubes (10 $\upmu \text {m}$ wall thickness) containing flowing blood with different oxygenation states. Figure 3 illustrates that MS-PAFI and DAS multispectral photoacoustic imaging provide the same spectral dependence for the absorption coefficient $\displaystyle \mu _\text{RBC}(\lambda ,{\mathbf{r}})$ in regions of interest visible on the two modalities. Consequently, quantitative SO$_2$ values are also identical, and allow discriminating the two capillary tubes based on blood oxygenation. Figure 3a illustrates that MS-PAFI can provide full-visibility quantitative images of SO$_2$, as opposed to DAS photoacoustic imaging for which only tube boundaries perpendicular to the probe axis are visible. Importantly, Fig. 3b also illustrates the absolute necessity to remove the noise background: black dots represent measurement values estimated without noise subtraction, which leads to a significantly overestimated value for the lower-oxygenation sample ($68\ \%$ as opposed to $52\ \%$).

### Quantitative SO$_2$ measurements in chicken embryo with MS-PAFI

**Figure 4:**
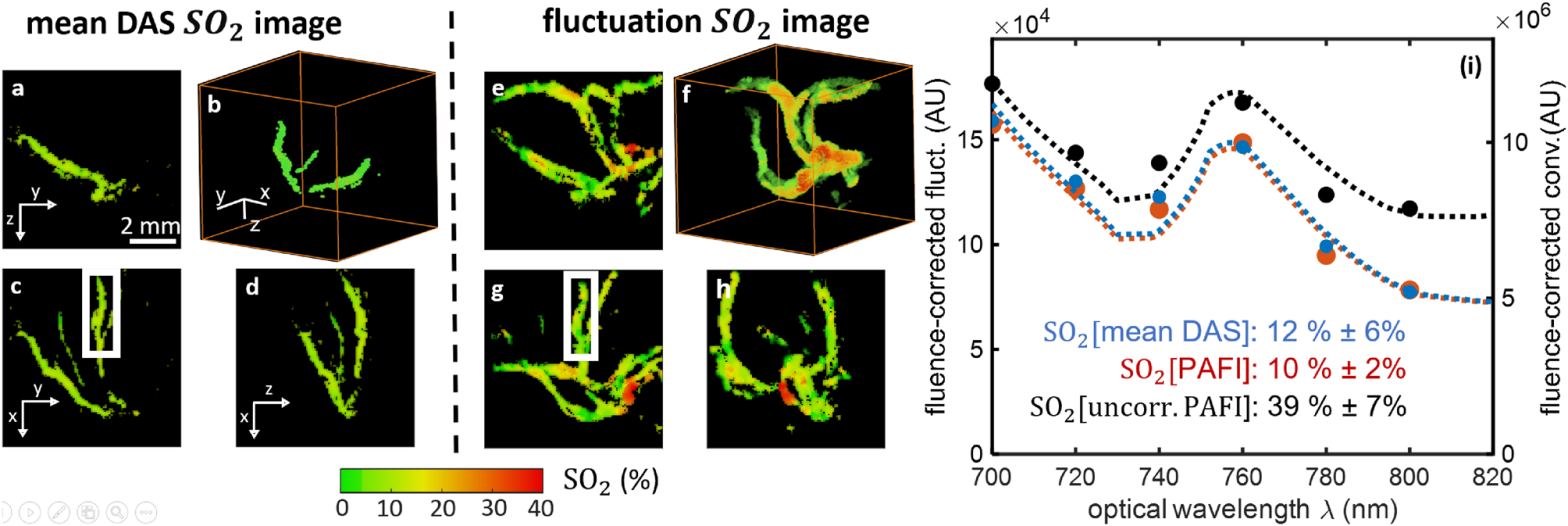
Three-dimensional quantitative SO$_2$ imaging in chicken embryo. (**a–d**) Visibility-limited SO$_2$ images obtained from conventional DAS multispectral photoacoustic imaging. (**e–h**) Full-visibility SO$_2$ images obtained from MS-PAFI. 2D images are maximum intensity projections through 3D data. (**i**) Relative spectral dependence of $\mu _\text{RBC}(\lambda ,{\mathbf{r}})$ estimated over the rectangular region of interest (ROI): blue, red and black estimates are respectively for DAS imaging, PAFI and uncorrected PAFI (without noise subtraction). ± values for SO$_2$ are the spatial standard deviation computed over the ROI.

We further demonstrate quantitative SO$_2$ imaging with MS-PAFI in chicken embryo (Fig. 4). Chicken embryo were used for their three-dimensional vascular structure with varying SO$_2$ oxygenation levels, to illustrate the visibility benefit of MS-PAFI over conventional multispectral imaging. As samples with negligible scattering, they were also ideal candidate to separate the visibility problem addressed here from the general challenge of local fluence measurements (out of the scope of this paper), a challenge common to all quantitative photoacoustic measurements, as already mentioned above. As for PAFI results shown in Fig. 2, 3D SO$_2$ images from MS-PAFI reveal the whole vasculature, as opposed to conventional DAS PA images corrupted by visibility artefacts. Following the approach introduced with capillary tubes, the validity of the approach is further confirmed by comparing SO$_2$ values in vessels visible on both PAFI and conventional DAS PA images, which were perfectly consistent (SO$_2[\text {MS-PAFI}]=12\pm 6\ \%$ and SO$_2[\text {DAS\ PA}]=10\pm 2\ \%$) as values spatially distributed within the region of interest shown in Fig. 4c,g. The absolute necessity for noise correction in order to obtain quantitative MS-PAFI measurements is further illustrated by the strong bias observed on the absorption spectra and by the strongly biased SO$_2$ value (SO$_2[\text {uncorrected\ MS-PAFI}]=39\pm 7\ \%$) obtained for the uncorrected case.

**Figure 5:**
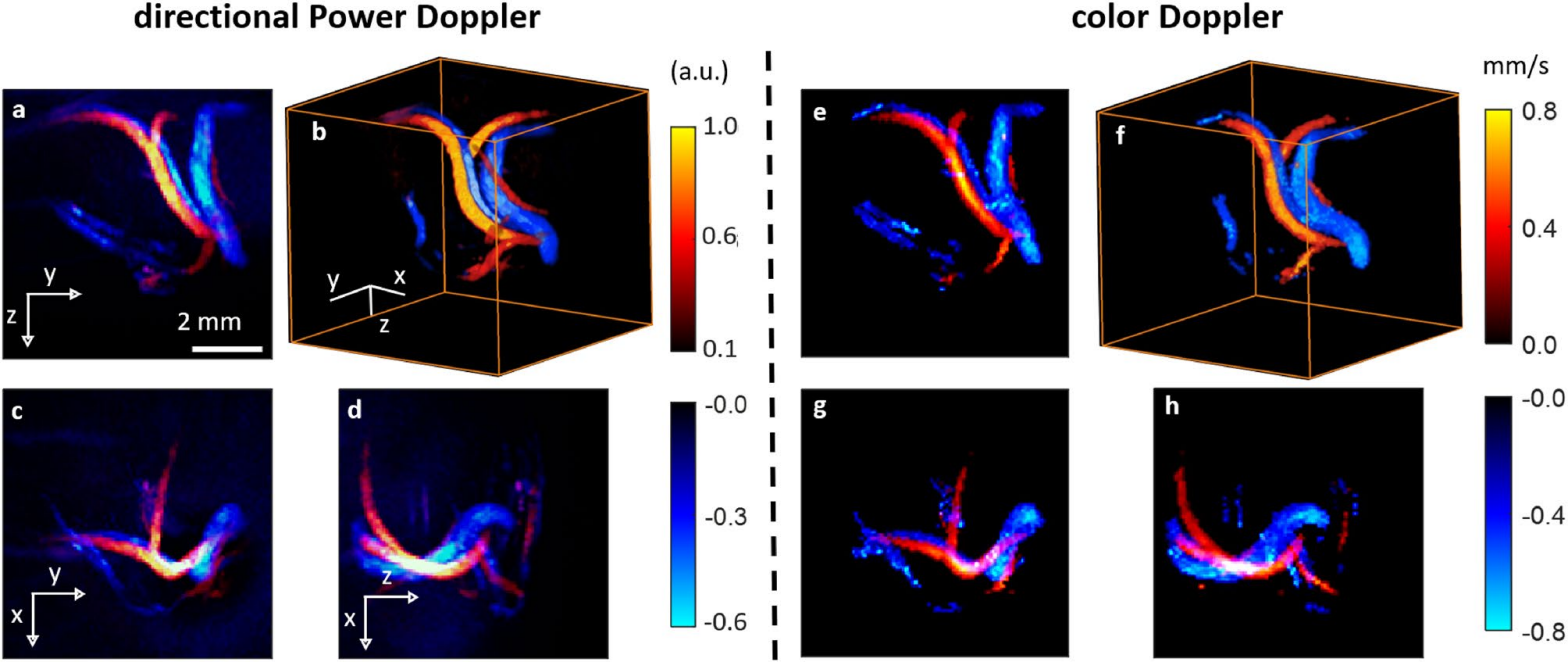
Flow dynamics in chicken embryo by ultrasound Directional Power Doppler (US-dPwD) and Color Doppler (US-CoD). Left panel (**a–d**) US-dPwD (red: upward flow, blue: downward flow). Right panel (**e–h**) US-CoD. (red: upward flow, blue: downward flow). 2D images are maximum intensity projections through the 3D data.

**Figure 6:**
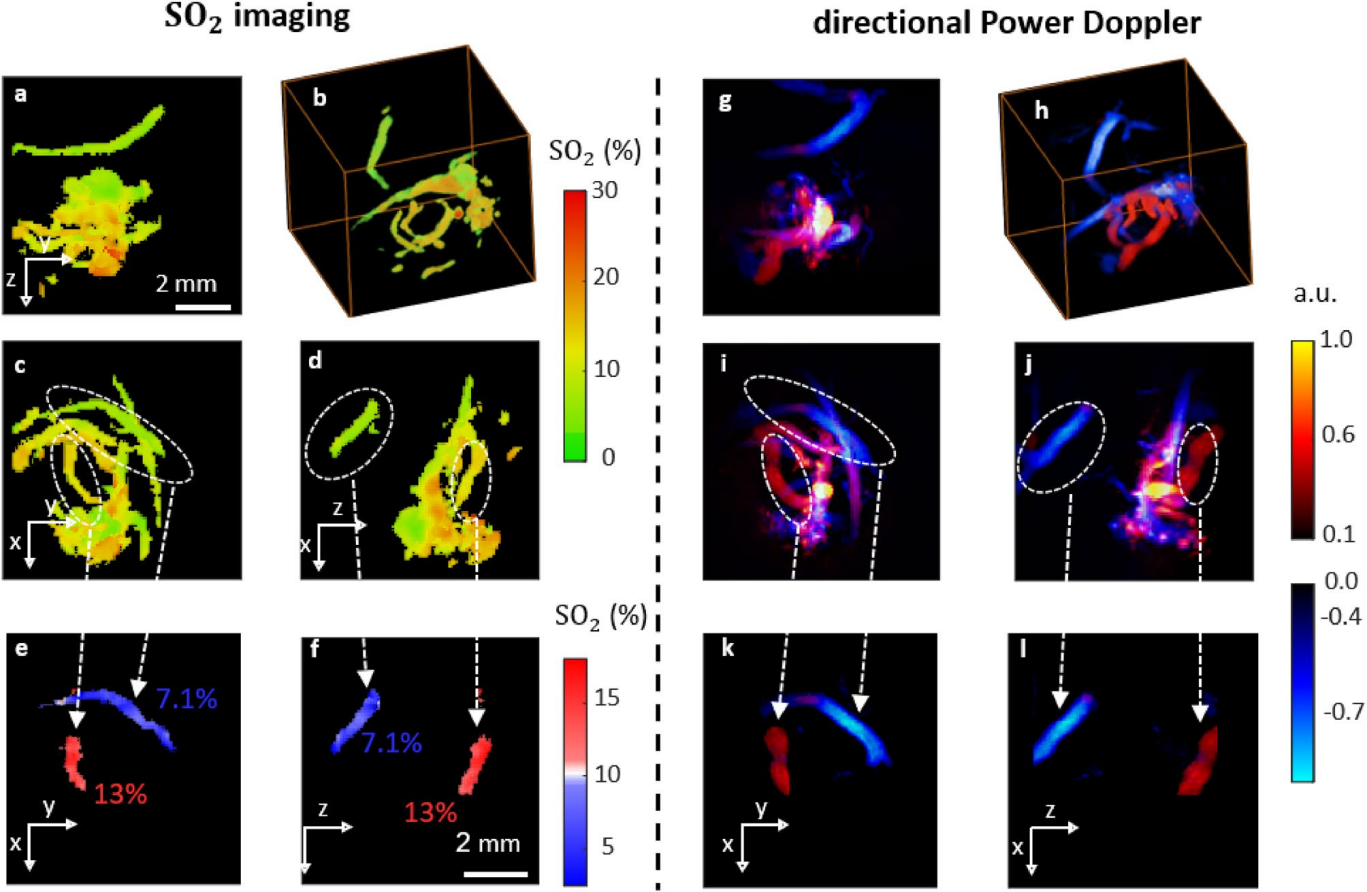
Simultaneous and complementary SO$_2$ imaging and Directional Power Doppler imaging in chicken embryo. 2D images are maximum intensity projections through the 3D data. Left panel (**a–f**) SO$_2$ imaging. (**e,f**) correspond to Maximum Intensity Projection images through ellipsoidal regions of interest shown in (**c,d**). The range and type of colormap for (**e,f**) has been optimized to highlight the difference in SO$_2$ values for two vessels with opposite flow directions. Right panel (**g–l**): Directional Power Doppler imaging. (**k,l**) correspond to Maximum Intensity Projection images through ellipsoidal regions of interest shown in (**i,j**), highlighting the difference in flow directions for each pair of vessels.

### Flow dynamics from ultrasound Doppler

In addition to the morphological information extracted from US-PwD (see Fig. 2), state-of-the-art ultrasound Doppler imaging techniques can also provide information about the blood flow direction (directional Power Doppler, US-dPwD) and speed (color Doppler, US-coD). Figure 5 illustrates the ability of our setup to provide US-dPwD and US-coD volumetric imaging simultaneously with MS-PAFI: while these two Doppler modalities are known, we implement them here for 3D imaging with only 256 elements and a commercial ultrasound electronics, whereas state-of-the-art demonstrations of 3D Doppler imaging required either mechanical scanning or 2D matrix with a large number of channels^21,41–44^. In the Supplementary Information, we further illustrate the ability to perform a time-resolved pulsed-Doppler analysis from the acquired data. As evidenced by Fig. 5, US-dPwD and US-coD allow separating venous from arterial flow based on the flow direction, a type of information unavailable from US-PwD or single-wavelength PAFI.

### Simultaneous and complementary SO$_2$ imaging and directional power Doppler imaging

The potential of the proposed method is fully exploited when analyzing conjointly SO$_2$ images and flow images from the Doppler modes, as illustrated by Fig. 6. PAFI and ultrasound Power Doppler provide similar morphological information; however, directional Power Doppler provides a straightforward discrimination between flow directions, while SO$_2$ allows quantifying the corresponding oxygenation. A specific analysis of SO$_2$ levels in the region of interest given in Fig. 6c–f confirms that vessels with opposite flow direction correspond to different oxygenation states (Fig. 6e,f).

## Discussion

By use of a sparse transducer with only 256 elements, connected to a commercially available 256-channel electronics, we have demonstrated both ultrasound and photoacoustic 3D imaging of the blood vasculature in chicken embryos, without contrast agents. We introduced multispectral photoacoustic fluctuation imaging (MS-PAFI), and demonstrated that it provides the same quantitative information as DAS photoacoustic imaging, with several major advantages: (1) MS-PAFI provides *full-visibility* images, (2) MS-PAFI provides images that are specific to blood, as the contributions from static chromophores are inherently filtered out by the approach, (3) PAFI is compliant with 3D imaging with a low number of transducers.

The very good sensitivity of our transducer, that allowed working with a small number of channels, comes from the relatively large size of the individual elements and their distribution over a spherical surface, at the cost of a limited field-of-view. A recent study implemented Power Doppler imaging in chicken embryos^45^ with a similar transducer geometry (although with a different technology and with 256 elements distributed over a plane), however ultrasound contrast agents were injected in the embryo to perform imaging, presumably because of sensitivity limitations. Ultrasound contrast agents were also used in the only prior publication reporting combined 3D ultrasound and 3D photoacoustic imaging^30^ but the authors chose the transcranial mouse brain model that probably led to a limited sensitivity and to the need for contrast agents. By implementing fluctuation imaging, for both photoacoustic and ultrasound data, we were able for the first time to provide simultaneously ultrasound Doppler and oxygenation 3D images of blood in vivo, without contrast agents.

The reconstruction of quantitative oxygenation value maps requires a quantitative measurement of $\mu (\lambda ,{\mathbf{r}})$, which in turn requires to compensate for the wavelength dependence of the local fluence $\Phi (\lambda ,{\mathbf{r}})$, as photoacoustic imaging is intrinsically sensitive to $\mu (\lambda ,{\mathbf{r}})\, \Phi (\lambda ,{\mathbf{r}})$. This is a general challenge of quantitative photoacoustic imaging, which has been the subject of intensive research over the past 20 years^12^. It was however out of the scope of this work to perform a full inverse problem that would include the reconstruction of $\Phi (\lambda ,{\mathbf{r}})$, as it is a whole problem in itself already present in conventional multispectral photoacoustic imaging. Accordingly, we thus performed proof-of-principle demonstrations for which the spectral dependence of $\Phi (\lambda ,{\mathbf{r}})$ could be derived directly from that measured at the sample surface, which is the case whenever the spectral dependence of the fluence is conserved through light propagation. The choice of the chicken embryo model was thus dictated not only by practical and ethical aspects, but also by this requirement that light could propagate towards the vascular networks with negligible spectral coloring effects. The fact that conventional multispectral photoacoustic imaging and MS-PAFI provide the same quantitative information (with enhanced visibility for MS-PAFI) was both predicted theoretically and demonstrated experimentally in our work, as one of its major objectives. A demonstration of quantitative SO$_2$ measurements with MS-PAFI inside scattering biological tissue (which requires solving the unknown fluence spectra challenge) was out of the scope of this work, which remained limited to a proof-of-concept demonstration performed in clear chicken embryo where the fluence could be calibrated. Additional concerns with in vivo scattering tissue are the overall penetration (sensitivity) of the technique, as fluence decays rapidly inside scattering tissue, and the robustness of the technique to tissue motion. These aspects were out of the scope of this manuscript.

Although the in vitro validation involved SO$_2$ values ranging from relatively low values (~ 50%) to relatively high values (~ 90%), we note that the SO$_2$ values measured in the embryo samples were relatively low on average, typically in the 5–20% range. As compared to the few oxygenation values that can be found in the literature for chicken embryos^46^, our values are in agreement with the low values reported for veins, but lower than the ones reported for arteries. We hypothesize that the chicken embryos were in a hypoxic state caused by the way the samples were imaged, with the chorioallantoic membrane (responsible for oxygen exchanges) immersed in water for acoustic coupling. Measuring regular arterial oxygenation values would require imaging through whole intact shells in air, which remains a challenge out of the scope of our work. Chicken embryos however provided a clear in vivo demonstration that even very slow capillary blood flows could yield quantitative measurements of the blood oxygenation, sensitive enough to discriminate small differences in oxygenation states, as illustrated by Fig. 6i,j.

As shown in the theoretical section, SVD processing is a key step to extract specifically the blood-induced fluctuations. Because the objectives of MS-PAFI is to provide quantitative oxygenation values, it is fundamental that the choice of the SVD filtering parameter (lower bound *a*) is made user-independent and has as little influence as possible in the derived oxygenation values. Accordingly, we used an automated method (detailed in the Supplementary information) to define *a* and derive robust oxygenation values, that agreed with those provided by conventional multispectral photoacoustic imaging with DAS reconstruction . As a major result of our study, we demonstrated that taking into account the electronic noise is fundamental to derive unbiased absorption spectra from multispectral photoacoustic fluctuation imaging, a key step to finally produce unbiased quantitative oxygenation values. As a key feature of MS-PAFI, its intrinsic specificity to hemoglobin makes the SO$_2$ inversion straightforward, without the need for spectral unmixing of other chromophores such as lipids, collagen and melanin required in conventional multispectral photoacoustic imaging^12,47^.

Because fluctuation imaging is inherently based on accumulating data over time, its main limitation is its low temporal resolution. Ultrasound Doppler can be performed with relatively short acquisition time, as the repetition rate can reach tens of kHz thanks to commercially available ultrafast electronics, compatible with ultrasound safety limits in tissue. In practice, optical tissue safety limits and the power limitations from the laser sources are the factors which fundamentally limits the maximum repetition rate for MS-PAFI, leading to acquisition time of several seconds to produce oxygenation images. Our system can however also provide multispectral single-shot imaging, which can be performed in real-time^25^ but at the cost of partial visibility. It thus offers the flexibility to trade between acquisition time and visibility for photoacoustic imaging. Model-based approaches could be implemented in the future and results compared to MS-PAFI ones. However, the optimal technological designs for both techniques might not be equivalent. Model-based approaches often suppose broadband detection/compensation to reconstruct multi-scale absorption profiles. Depending on the target size, this may impose a tradeoff in the choice of the central frequency: the increase of the central frequency at frequencies typically higher than 5 or 8 MHz results in a poor sensitivity at low frequencies, preventing a proper reconstruction of large vessels. This limitation happens at the cost of spatial resolution. In contrast, MS-PAFI works with native limited-bandwidth detectors and the frequency can be increased without loss of multi-scale information, which could have benefits for high-resolution multi-scale imaging of vasculature.

In conclusion, we proposed an approach that can easily be implemented on standard 256-channels electronics and provides a unique comprehensive characterization of the blood vasculature, based on the synergistic combination of ultrasound and photoacoustic 3D quantitative imaging conjointly enabled via fluctuation imaging.

## Materials and methods

### Ultrasound transducer

The ultrasound transducer is a 256-channel matrix array designed in our laboratory and fabricated by Imasonic (Voray-sur-l’Oignon, France). The surface of the transducer is a spherical shell with a 10-mm diameter hole on its main axis (z-axis). The individual elements are circular elements with diameter $d=2$ mm, distributed over a Fermat spiral with a divergence angle of $137.51^{\circ }$ (golden angle), leading to the sunflower pattern shown in Fig. 1c. This specific pattern was first proposed by Martinez and colleagues in the context of ultrasound imaging^35^, and is known to minimize grating lobes^35,48^ while maximizing the packing of circular elements^49^. Our transducer was first used in our earlier work to obtain preliminary results demonstrating 3D PAFI^31^, but was never described before. The radius of curvature (focal distance F) and the outer diameter D of the array are $F = 35$ mm and $D =50$ mm respectively, corresponding to a F-number = $F/D=0.7$. The center frequency of the probe is $f_c=8$ MHz, with a -6dB relative bandwidth (one-way) of $80\ \%$. The geometrical parameters of the probe (including *F*, *d* and *D*) result from several compromises between resolution, field-of-view and detection sensitivity, for a given number (256) of elements. The corresponding field of view for both PA and US imaging is located at the center of curvature of the array, and overs approximately a $8\ \text{mm}\times 8\ \text{mm} \times 8\ \text{mm}$ volume. An in-house coupling cone allowed to fill the volume between the transducer surface and the sample with degassed deionized water. A latex membrane was attached at the tip of the cone to ensure coupling between the water and the sample. By controlling the amount of water inside the cone, the latex membrane can be inflated/deflated to adjust the imaging area while preserving acoustic coupling. When the latex membrane is flat, the imaging location is centered at a 10.5 mm depth, i.e. the imaging depth ranges from 6.5 to 14.5 mm. PSFs were measured experimentally and resolutions were evaluated from the full width at half maximum on each reconstructed profiles. At focus, respectively for PA and US imaging, the lateral resolution is 0.18 mm and 0.12 mm and the axial resolution is 0.27 mm and 0.22 mm. This resolution decays away from focus, for example, respectively for PA and US imaging at the coordinate [2; 2; 3] mm, the lateral resolution is 0.28 mm and 0.16 mm and the axial resolution is 0.35 mm and 0.23 mm. Note that we can estimate the resolution of PAFI and power Doppler by dividing those numbers by $\sqrt{2}$ as indicated by the SOFI theory^39^.

### Instrumentation

The transducer was connected to a commercially available 256-channel ultrasound electronics (Vantage HF 256, Verasonics, USA), capable of both emission and reception of 256 ultrasound signals in parallel, with repetition rates up to several kHz. For all experiments, the signals were digitized with a sample rate of 31.25 MHz. The laser source for the photoacoustic modality is an optical parametric oscillator (OPO) pumped by a diode-pumped Nd:YAG laser (Spitlight, Innolas, Germany, 100 Hz repetition frequency, 5-ns pulses), with a wavelength tunable for each individual laser pulse (range 680–900 nm). The output beam was coupled to the probe through a custom fiber bundle (Ceramoptec, Germany), attached to the transducer through its central hole (Fig. 1a). A photodiode was used to monitor the energy of each laser pulse $E_k(\lambda )$ at the output of the laser. The photodiode signal is integrated with a boxcar gated integrator (Standford Research System, SR 250) and digitized by a USB acquisition board (TiePie, HS6). A prior precalibration step was performed to rlink the energy of each laser pulse $E_k(\lambda )$ to the peak fluence $\Phi ^\text{sample}_k(\lambda )$ measured at the surface of the sample, taking into account all possible losses and wavelength dependences induced by fiber coupling, fiber transmission and illumination geometry. The fluence at the sample surface ranged from ${\overline{\Phi }}^\text{sample}(\lambda =880\ \text{nm})=2.5\ \text{mJ}/\text{cm}^2$ to ${\overline{\Phi }}^\text{sample}(\lambda =720\ \text{nm})=8\ \text{mJ}/\text{cm}^2$, corresponding to $[250-800]\ \text{mW}/\text{cm}^2$ in terms of temporal-average fluence rate. The synchronisation between the ultrasound electronics and the laser was implemented via a delay generator, and was designed to minimize the temporal jitter between the laser shots and the ultrasound acquisition. The ultrasound electronics acts as the master, triggering laser shots via the Pockels cell of the slave laser at 100 Hz. A bypass circuit was provided by Innolas to reduce the laser temporal jittering down to a typical value of $\pm 1$ ns, in response to the external trig signal from the ultrasound electronics. We verified experimentally and via simulations that such a low jitter value had a negligible influence on photoacoustic fluctuation imaging with a center frequency of 8 MHz, as compared to other fluctuations sources (including the laser pulse energy fluctuations, the fluctuations of interest from the blood flow and the electronic noise), and it was consequently discarded in this work.

### Samples preparation and positioning

The chicken embryo (fertilized eggs) were obtained from a local farm and placed in an incubator for 9 days. After making a small hole through the shell by use of a scalpel, 1.5 mL of egg white was removed with a syringe and the top part of the shell was cut with scissors. Warm PBS was added on the top of the sample to ensure acoustic contact with the transducer coupling cone without touching the membrane of the embryo. The sample was held on a hammock made with a plastic foil, attached to a bowl containing water kept at 36 $^\circ$C using a hot plate (Fig. 1a). Pre-positioning the sample relatively to the probe was first achieved using real-time cross-sectional pulse-echo ultrasound images (YZ, XZ and XY planes) with a display rate of 10 Hz, based on the echoes from the sample boundaries. Fine positioning was then achieved through 3D single-shot DAS photoacoustic imaging of the blood vasculature, based on maximum intensity projections (MIP) of 3D photoacoustic images displayed at 1 Hz.

**Figure 7:**
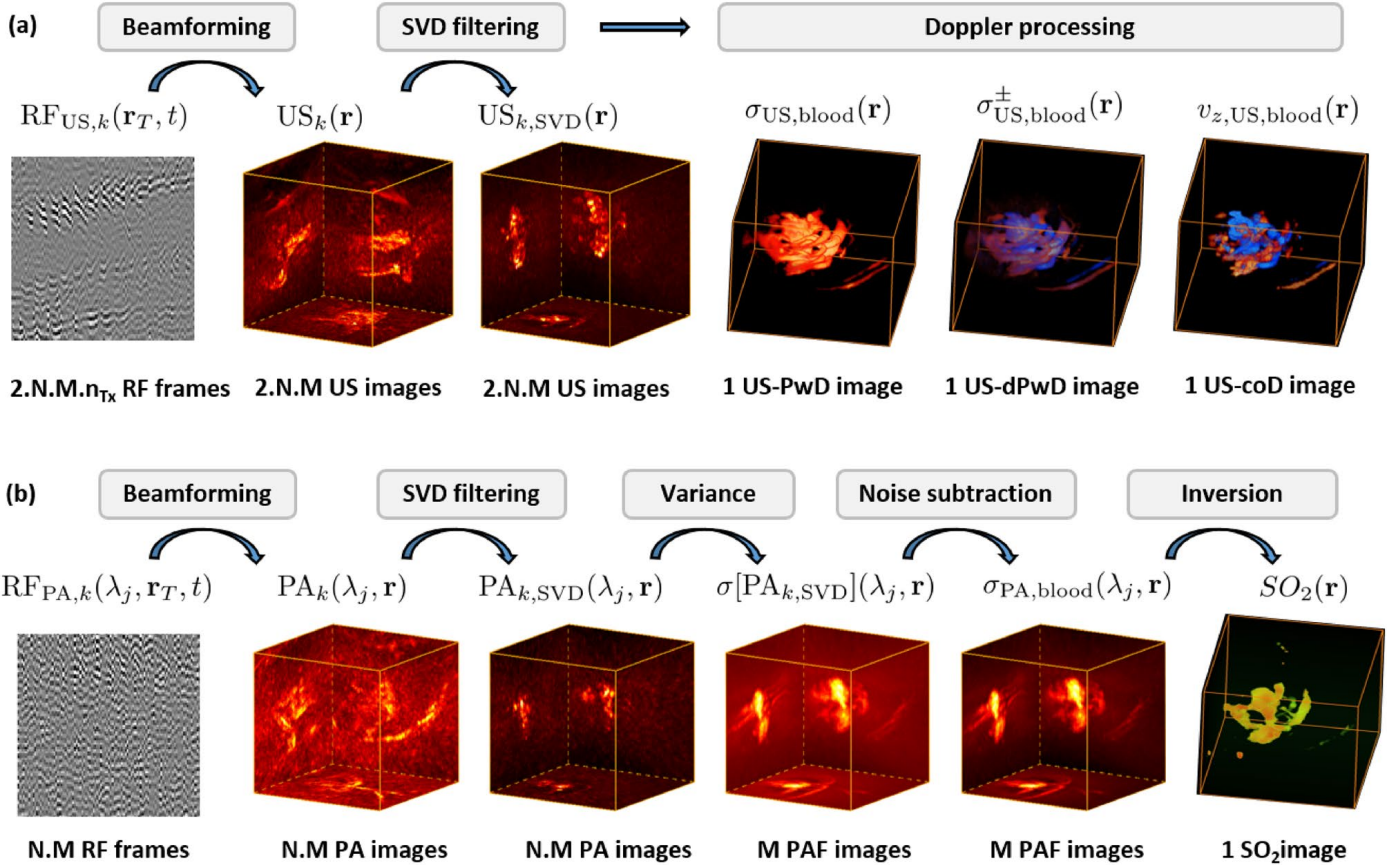
Data processing steps from radiofrequency signals (RF) to Doppler images (**a**) and photoacoustic fluctuation (PAF) images (**b**), applied to N individual sequences as defined in Fig. 1b. ${\mathbf{r}}_T$ refers to the coordinates of each transducer element (RF frames) and ${\mathbf{r}}$ refers to the coordinates of the beamformed images. multispectral PAFI produces M different PAF images, one per wavelength, from which one final SO$_2$ image is computed.

### Data acquisition

The acquired radio-frequency (RF) data for a complete measurement corresponded to N repeated sequences of interleaved ultrasound and photoacoustic acquisitions, as represented schematically in Fig. 1c. One elementary set of RF data, consisting of $n_t \times n_\text{elts}$ temporal points times the number of transducer elements ($n_\text{elts}=256$), corresponded to one laser shot for photoacoustic data, and to the emission by one single element for ultrasound data. For ultrasound imaging, the waveforms defined by one single period at the center frequency were driven at 40 V. Three-dimensional ultrasound images were computed by compounding the 3D images corresponding to $n_{T_x}=10$ individual ultrafast ultrasound acquisitions (repetition rate 17.8 kHz, i.e. 56 $\upmu$s between successive acquisitions), following the principles of ultrafast imaging with diverging waves^50^. Each of these $n_{T_x}$ acquisitions corresponded to the emission of ultrasound by one of the $n_{T_x}=10$ elements depicted in Fig. 1c (red stars). For each of the N sequences, $M=10$ sets of photoacoustic RF data, corresponding to *M* wavelengths, were acquired at 100 Hz (limited by the laser repetition rate) in an interleaved manner with ultrasound RF data. Ultrafast ultrasound sequences of $n_{T_x}$ acquisitions (0.56 ms total duration each) were repeated every 5 ms (200 Hz repetition rate), twice faster that photoacoustic acquisitions. In principle, ultrafast sequences could have been acquired with a repetition rate higher than 200 Hz, but at the cost of more data to handle and process. 200 Hz turned out to be sufficient to perform quantitative Doppler measurement in chicken embryo without significant aliasing. In summary, a complete acquisition resulted in a set of $N\times M$ multispectral photoacoustic RF dataset (100 Hz rate) and $N\times 2\times M$ ultrafast ultrasound sequences (200 Hz rate). For all the results presented in this work, the number N of sequences was of the order of a few hundreds, corresponding to a total acquisition time of the order of a few tens of seconds (10-s acquisition time for N = 100).

### Data processing

The various data processing steps leading to 3D ultrasound Doppler and photoacoustic fluctuation images from RF data are summarized in Fig. 7. All data processing steps were performed with Matlab 2020 (https://www.mathworks.com/), which was also used to drive all the instruments.

#### Image reconstruction

All acquired real-valued RF signals were transformed into complex-valued RF signals through a Hilbert transform, before further processing. For both modalities, standard delay-and-sum beamforming was implemented to reconstruct complex-valued 3D images from each elementary set of RF data. The delay-and-sum (DAS) algorithm used in this study consists in summing the complex-valued Hilbert-transformed signal along curves given by theoretical time-of-flights, using a phase rotation approximation at the probe central frequency for interpolation, such as performed by Bertolo and colleagues for instance^51^. No apodization was applied. One 3D ultrasound image $\text{US}_k({\mathbf{r}})$ was obtained for each ultrafast ultrasound sequence, by compounding (i.e. coherently summing) the $n_{T_x}$ beamformed 3D images, and one 3D photoacoustic image $\text{PA}_k(\lambda ,{\mathbf{r}})$ was obtained for each laser shot. We note that for photoacoustic acquisition, parasitic electromagnetic radiation from the laser could sometimes be observed on the RF signals, as a pulsed signal identical on all channels: such transducer-independent parasitic signals were filtered out by subtracting, to all the 256 signals of a given laser shot, the signal averaged over all the 256 transducers. All conventional DAS photoacoustic and ultrasound images in the manuscript correspond to the temporal averaging of 3D DAS images before SVD filtering. For photoacoustic imaging, this average is performed separately for each wavelength, leading to *M* estimates of $\displaystyle E[\text{PA}_k](\lambda ,{\mathbf{r}})$.

#### Singular-value-decomposition (SVD) filtering

Out of the maximum numbers of 3D images available from a complete measurement of N sequences, the numbers $N_\text{PA}$ and $N_\text{US}$ of single-shot photoacoustic and ultrasound images, exploited and processed to produce respectively photoacoustic fluctuation images and ultrasound Doppler images, are given in the supplementary information (table S1) for each results shown in the manuscript, with $N_\text{PA}\le N\times M$ and $N_\text{US}\le N\times 2 \times M$. For both modalities, a singular-value-decomposition (SVD) filtering was first applied to the stack of exploited 3D images ($N_\text{PA}$ for photoacoustic fluctuation imaging, $N_\text{US}$ for ultrasound Doppler), following the approach introduced in earlier works in both ultrasound and photoacoustic imaging^31,36^. For photoacoustic imaging, a single SVD filtering step was applied to the whole stack of $N_\text{PA}$ images for all wavelengths at once, as this turned out to be more efficient than applying SVD-filtering for each wavelength separately. From a computational perspective, we note that this SVD step is the only additional step as compared to conventional DAS beamforming. For the sake of clarity, we here briefly recall the principles of SVD filtering, further detailed in the Supplementary Information: SVD decomposes a temporal series of 3D images into a basis of spatio-temporal singular vectors, with a weight given by singular values. Fluctuations with different spatio-temporal patterns may thus be separated when decomposed on such a basis. For both ultrasound and photoacoustic fluctuation imaging, the fluctuations from blood flow may be selectively extracted by keeping only singular vectors with index above some lower bound *a*, after the singular vectors have been sorted by decreasing singular values. For both modalities, filtering out singular vectors with index smaller than the lower bound *a* turns out to remove dominant spatially coherent parasitic fluctuations (including tissue motion for ultrasound Doppler^36^ and laser pulse energy fluctuation for PAFI^31^). Because PAFI involves additional laser-induced fluctuations, as compared to ultrasound Doppler, *a* has to be chosen specifically for each modality. In ultrasound Doppler imaging, the choice of *a* is usually determined empirically based on a qualitative assessment of the image quality, which is the approach that we implemented to produce all the Doppler images shown in our work. For MS-PAFI, because we propose a novel method to produce quantitative SO$_2$ values, care was taken to ensure that the choice of *a* allows efficiently extracting the fluctuations of interest while producing user-independent SO$_2$ values (see Supplementary Information). Table S1 in the Supplementary material provides the number of images ($N_\text{PA}$ or $N_\text{US}$) and lower bound *a* used for all the results presented in this work.

#### Fluctuation imaging

Estimation of the fluctuation images were computed from the stacks of SVD-filtered images. The processing steps to obtain ultrasound Doppler images are standard ones, briefly summarized in the Supplementary Information along with relevant references. For photoacoustics, PAFI images are computed for each wavelength via the standard deviation of the stack of images along the temporal dimension, as for ultrasound Power Doppler. However, as explained in the section presenting the principles of MS-PAFI, the standard deviation due to blood flow specifically was estimated after first subtracting the variance of the electronic noise $\sigma ^2_{n}$, as $\displaystyle \sigma _{\text{PA,blood}}(\lambda ,{\mathbf{r}})=\sqrt{\sigma ^2[\text{PA}_{k,\text{SVD}}](\lambda ,{\mathbf{r}}) - \sigma ^2_{n}}$. The method to estimate $\sigma ^2_{n}$ is detailed in the Supplementary Information.

#### Quantitative SO$_2$ imaging

By use of Eq. (4), the photoacoustic fluctuation image provides access to the absorption coefficient through

$$\begin{aligned} \displaystyle \mu _\text{RBC}(\lambda ,{\mathbf{r}})=\frac{\sigma _{\text{PA,blood}}(\lambda ,{\mathbf{r}})}{{\overline{\Phi }}(\lambda ,{\mathbf{r}}) \, K_{\sigma _{\text{PA,blood}}}({\mathbf{r}})}=\frac{1}{ K_{\sigma _{\text{PA,blood}}}({\mathbf{r}})}\, \frac{\sqrt{\sigma ^2[\text{PA}_{k,\text{SVD}}](\lambda ,{\mathbf{r}}) - \sigma ^2_{n}}}{{\overline{\Phi }}(\lambda ,{\mathbf{r}})}, \end{aligned}$$

with $\displaystyle K_{\sigma _{\text{PA,blood}}}({\mathbf{r}})=\Gamma ({\mathbf{r}})\,\sqrt{\eta ({\mathbf{r}}) \, W[\eta ({\mathbf{r}})] \, V_\text{RBC}({\mathbf{r}})\, \left[ f_\text{vessels}\times |h_{\text{PA}}|^2\right] ({\mathbf{r}})}$. A quantitative *absolute* estimation of the absorption coefficient is not possible as it would require the knowledge of $K_{\sigma _{\text{PA,blood}}}({\mathbf{r}})$, in addition to the local fluence $\Phi (\lambda ,{\mathbf{r}})$. However, the information on the local oxygenation value is carried only through the wavelength-dependence of $\mu _\text{RBC}(\lambda ,{\mathbf{r}})$, through the following definition of SO$_2$:

$$\begin{aligned} \mu _\text{RBC}(\lambda ,{\mathbf{r}},SO_2)=C_\text{tot,Hb}({\mathbf{r}})\, \mu _\text{tot,Hb}(\lambda ,{\mathbf{r}},SO_2)=C_\text{tot,Hb}({\mathbf{r}})\,\big [ SO_2({\mathbf{r}})\, \mu _{{\text{HbO}}_{2}}(\lambda )+[1-SO_2({\mathbf{r}})]\, \mu _\text{Hb}(\lambda )\big ], \end{aligned}$$

where $C_\text{tot,Hb}({\mathbf{r}})$ is the total molar concentration of haemoglobin in the RBC at position ${\mathbf{r}}$, and $\mu _{{\text{HbO}}_{2}}$ and $\mu _\text{Hb}$ are respectively the molar absorption coefficients of oxy- and deoxyhaemoglobin. It is thus possible to estimate $SO_2({\mathbf{r}})$ by fitting at each position ${\mathbf{r}}$ the measured quantity $\displaystyle \frac{\sqrt{\sigma ^2[\text{PA}_{k,\text{SVD}}](\lambda ,{\mathbf{r}}) - \sigma ^2_{n}}}{{\overline{\Phi }}(\lambda ,{\mathbf{r}})}$ to $\displaystyle \kappa ({\mathbf{r}}) \, \mu _\text{tot,Hb}(\lambda ,{\mathbf{r}},SO_2)$, where $\kappa ({\mathbf{r}})$ and $SO_2({\mathbf{r}})$ are the fitted parameters, given models for $\mu _{{\text{HbO}}_{2}}(\lambda )$ and $\mu _\text{Hb}(\lambda )$. The values used for $\mu _{{\text{HbO}}_{2}}(\lambda )$ and $\mu _\text{Hb}(\lambda )$ at each measured wavelength, taken from a reference website^52^, are provided in the Supplementary Information. The exact same approach is used for conventional SO$_2$ imaging with the single-shot/mean image instead of the fluctuation image, and in both case, the wavelength-dependence of $\Phi (\lambda ,{\mathbf{r}})$ is required in order to fit data to the model for the absorption coefficient of total haemoglobin. When this wavelength dependence is unaffected by propagation (i.e. no spectral coloring due to propagation), $\Phi (\lambda ,{\mathbf{r}})$ may be written as ${\overline{\Phi }}(\lambda ,{\mathbf{r}})={\overline{\Phi }}^\text{sample}(\lambda )\, \xi ({\mathbf{r}})$, where ${\overline{\Phi }}^\text{sample}(\lambda )$ is the maximum fluence at the sample surface and $\xi ({\mathbf{r}})$ describes the wavelength-independent propagation of the fluence through the sample. The knowledge of $\xi ({\mathbf{r}})$ is not needed to derive SO$_2$ values, as the fit is performed locally at each position ${\mathbf{r}}$, and $\xi ({\mathbf{r}})$ can be included in $\kappa ({\mathbf{r}})$. Under this assumption for quasi-transparent chicken embryo samples, we thus estimated the $S0_2$ values at each point in space through the following 2-parameter minimization process:

5 $$\begin{aligned} {[}SO_2({\mathbf{r}}),\kappa ({\mathbf{r}})]=\underset{SO_2({\mathbf{r}}),\kappa ({\mathbf{r}})}{\text{argmin}} \sum _{\lambda _j}\left|\frac{\sqrt{\sigma ^2[\text{PA}_{k,\text{SVD}}](\lambda _j,{\mathbf{r}}) - \sigma ^2_{n}}}{{\overline{\Phi }}^\text{sample}(\lambda _j)}-\kappa ({\mathbf{r}})\, \left[ SO_2({\mathbf{r}})\, \mu _{{\text{HbO}}_{2}}(\lambda _j)+[1-SO_2({\mathbf{r}})]\, \mu _{{\text{Hb}}_{2}}(\lambda _j)\right] \right|^2. \end{aligned}$$

The minimization was implemented by use of the *fminsearch* Matlab function. All the wavelength-dependent curves for the optical absorption coefficient shown in the manuscript correspond to $\displaystyle \frac{\sqrt{\sigma ^2[\text{PA}_{k,\text{SVD}}](\lambda _j,{\mathbf{r}}) - \sigma ^2_{n}}}{{\overline{\Phi }}^\text{sample}(\lambda _j)}$ for the experimental data and to $\displaystyle \kappa ({\mathbf{r}})\, \left[ SO_2({\mathbf{r}})\, \mu _{{\text{HbO}}_{2}}(\lambda _j)+[1-{\text {SO}}_2({\mathbf{r}})]\, \mu _{{\text{Hb}}_{2}}(\lambda _j)\right]$ resulting from the minimization process. Depending on the signal-to-noise ratio, which decreases with increasing wavelength, the number of wavelengths used to perform the fit varied from 7 (Fig. 4) to 10 (Figs. 3, 6). Optimizing the number and values of measured wavelengths was out of the scope of our study, but would be useful to minimize the total acquisition time. It was also out of our scope to further exploit the fitted parameter $\kappa ({\textbf{r}})$ which gives an estimation of $C_\text{tot,Hb}({\textbf{r}})\, K_{\sigma _{\text{PA,blood}}}({\textbf{r}})\, \xi (r)=C_\text{tot,Hb}({\textbf{r}})\, \Gamma ({\textbf{r}}) \, \sqrt{\eta ({\textbf{r}}) \, W[\eta ({\textbf{r}})] \, V_\text{RBC}({\textbf{r}})\, \left[ f_\text{vessels}\times |h|^2\right] ({\textbf{r}})} \, \xi (r)$. We note that this quantity may however be potentially useful for further quantitative analysis in the framework of photoacoustic fluctuation imaging.

#### Results visualization

Three-dimensional data were visualized either through maximal intensity projections (MIP) computed with Matlab 2020 (https://www.mathworks.com/) or 3D rendering produced with AMIRA 6.7 (https://www.thermofisher.com/fr/fr/home/electron-microscopy/products/software-em-3d-vis/amira-software.html). For non quantitative images such as in the Fig. 2, colorbars ranged between the minimum and the maximum of each 3D images. SO$_2$ values in 2D images are computed at the pixels corresponding to the MIP through the fluctuation image (computed at the wavelength leading to the strongest signal-to-noise ratio). In addition, a gaussian mask, computed from a 2D gaussian filtering of the binarized MIP image (with a width of 1.5 pixels), was applied to the 2D SO$_2$ map to smooth the borders of the vessels. 3D SO$_2$ maps were interpolated before 3D rendering. For color Doppler and directional Power Doppler, which involves signed values, a “hot” colormap and a “cold” colormap were used respectively for the positive and negative values, merged via a transparency effect to produce the final image.

## Supplementary Information

Supplementary Information.

## Data Availability

Example codes and datasets are available from the corresponding author on reasonable request.
