# Supplementary material for: Full-visibility 3D imaging of oxygenation and blood flow by simultaneous multispectral photoacoustic fluctuation imaging (MS-PAFI) and ultrasound Doppler
Source: Sci Rep. 2023 Feb 20;13:2961. doi: 10.1038/s41598-023-29177-9 (PMC9941110; doi:10.1038/s41598-023-29177-9)
Supplement: Supplementary file 1 — Supplementary Information. [file 41598_2023_29177_MOESM1_ESM.pdf]

# Full-visibility 3D imaging of oxygenation and blood flow by simultaneous multispectral photoacoustic fluctuation imaging (MS-PAFI) and ultrasound Doppler : Supplementary Information

**Guillaume Godefroy[1], Bastien Arnal[1], and Emmanuel Bossy[1,*]**

[1]Univ. Grenoble Alpes, LIPhy, CNRS, Grenoble, 38000, France
[*]emmanuel.bossy@univ-grenoble-alpes.fr

## ABSTRACT

This supplementary information provides technical details on theoretical and experimental aspects of the main manuscript. We first provide a derivation of Eq. 3, which constitutes a major theoretical result of our work. In a second part, we recall in details the principles of singular-value-decomposition, from which we then derive a method for the SVD-dependent noise compensation required for quantitative $SO_2$ imaging. We further discuss the influence and choice of the lower bound $a$ for the SVD filtering step. For readers not already familiar with various ultrasound Doppler techniques, we detail how the various type of Doppler images are computed from temporal stacks of ultrasound images, and we provide an example of a pulsed Doppler analysis. Finally, we provide a list of parameters used to produce all the images shown in the manuscript.

## Contents

**1 Derivation of the photoacoustic fluctuation image in presence of laser fluctuations and electronic noise** **1**

**2 Singular-value-decomposition (SVD) filtering** **2**

2.1 Principles . . . . . 2

2.2 Noise compensation for multispectral PAFI . . . . . 3

2.3 Influence of the lower bound $a$ . . . . . 3

2.4 Automated determination of the lower bound $a$ . . . . . 3

**3 Ultrasound Doppler** **5**

3.1 Data processing . . . . . 5

3.2 An example of pulsed Doppler analysis . . . . . 6

**4 Tables of parameters** **7**

**References** **7**

## 1 Derivation of the photoacoustic fluctuation image in presence of laser fluctuations and electronic noise

In this section, we provide a demonstration for Eq. 3 of our manuscript. Under the assumption that both the light fluence and all the physical parameters of blood are constant at the scale of the PSF, but may vary from one vessel to another, the theoretical expressions for the single photoacoustic image $\mathrm{PA}_k(\mathbf{r})$, the conventional/mean photoacoustic image $m_{\mathrm{PA}} = \mathrm{E}[\mathrm{PA}_k]$ and the photoacoustic fluctuation image $\sigma_{\mathrm{PA}} = \sigma[\mathrm{PA}_k] = \sqrt{\mathrm{E}[\mathrm{PA}_k^2] - \mathrm{E}[\mathrm{PA}_k]^2}$ provided in our previous work[1] can be readily adapted to take into account the wavelength and spatial dependence of all relevant physical properties:

$$\mathrm{PA}_k(\lambda,\mathbf{r}) = \Gamma(\mathbf{r}) \times \mu_{\mathrm{RBC}}(\lambda,\mathbf{r}) \times \Phi(\lambda,\mathbf{r}) \times \left|(g_k \times f_{\mathrm{vessels}}) * h\right|(\mathbf{r}) \tag{S1a}$$

$$m_{\mathrm{PA}}(\lambda,\mathbf{r}) = \Gamma(\mathbf{r}) \times \mu_{\mathrm{RBC}}(\lambda,\mathbf{r}) \times \Phi(\lambda,\mathbf{r}) \times \eta(\mathbf{r}) \times \left|f_{\mathrm{vessels}} * h\right|(\mathbf{r}) \tag{S1b}$$

$$\sigma_{\mathrm{PA}}(\lambda,\mathbf{r}) = \Gamma(\mathbf{r}) \times \mu_{\mathrm{RBC}}(\lambda,\mathbf{r}) \times \Phi(\lambda,\mathbf{r}) \times \sqrt{\eta(\mathbf{r}) \times W[\eta(\mathbf{r})] \times V_{\mathrm{RBC}}(\mathbf{r}) \times [f_{\mathrm{vessels}} * |h|^2]\,(\mathbf{r})} \tag{S1c}$$

The expression above are valid only when blood is the sole source of fluctuation. We now further extend the theory by taking into account fluctuations from the laser fluence and from the electronics. We assume that the electronic noise yields a stationary random background $n_k(\mathbf{r})$ on the beamformed images, with $\mathrm{E}[n_k(\mathbf{r})] = 0$ and $\mathrm{E}[|n_k(\mathbf{r})|^2] = \sigma_n^2$. The fluence fluctuations are described by $\Phi_k(\lambda,\mathbf{r}) = \overline{\Phi}(\lambda,\mathbf{r})[1+\delta_k(\lambda)]$, where $\overline{\Phi}(\lambda,\mathbf{r}) = \mathrm{E}[\Phi(\lambda,\mathbf{r})]$ is the mean local fluence and $\delta_k(\lambda)$ the *relative* laser pulse energy fluctuation. By definition $\mathrm{E}[\delta_k(\lambda)] = 0$, and we further note $\sigma^2_{\mathrm{laser}}(\lambda) = \mathrm{E}[\delta_k^2(\lambda)]$ . A single photoacoustic image $\mathrm{PA}_k(\mathbf{r})$ may now be expressed as $\mathrm{PA}_k(\lambda,\mathbf{r}) = \Gamma(\mathbf{r}) \times \mu_{\mathrm{RBC}}(\lambda,\mathbf{r}) \times \overline{\Phi}(\lambda,\mathbf{r}) \times [1+\delta_k(\lambda)] \times \left|(g_k \times f_{\mathrm{vessels}}) * h\right|(\mathbf{r}) + n_k(\mathbf{r})$, which can be rewritten as

$$\mathrm{PA}_k(\lambda,\mathbf{r}) = \mathrm{PA}_{k,\mathrm{blood}}(\lambda,\mathbf{r}) \times [1+\delta_k(\lambda)] + n(\mathbf{r}) = \mathrm{PA}_{k,\mathrm{blood}}(\lambda,\mathbf{r}) + \delta_k(\lambda) \times \mathrm{PA}_{k,\mathrm{blood}}(\lambda,\mathbf{r}) + n_k(\mathbf{r}) \tag{S2}$$

where $\mathrm{PA}_{k,\mathrm{blood}}(\lambda,\mathbf{r}) = \Gamma(\mathbf{r}) \times \mu_{\mathrm{RBC}}(\lambda,\mathbf{r}) \times \overline{\Phi}(\lambda,\mathbf{r}) \times \left|(g_k \times f_{\mathrm{vessels}}) * h\right|(\mathbf{r})$ is the noise free image for a constant fluence $\overline{\Phi}(\lambda,\mathbf{r})$. Assuming that the electronic noise, the laser fluctuations and the blood fluctuations are independent stochastic variables, the variance photoacoustic image $\sigma^2[\mathrm{PA}_k](\lambda,\mathbf{r})$ is given by the sum of the variance of each term. Moreover, using independence again and $\mathrm{E}[\delta_k] = 0$, the variance of the second term of may further be expressed as

$$\sigma^2[\delta_k \times \mathrm{PA}_{k,\mathrm{blood}}] = \mathrm{E}[\delta_k^2] \times \mathrm{E}[\mathrm{PA}^2_{k,\mathrm{blood}}] = \mathrm{E}[\delta_k^2] \times (\sigma^2[\mathrm{PA}_{k,\mathrm{blood}}] + \mathrm{E}^2[\mathrm{PA}_{k,\mathrm{blood}}])$$

Given $m_{\mathrm{PA}} = \mathrm{E}[\mathrm{PA}_{k,\mathrm{blood}}]$, and using the notation $\sigma^2_{\mathrm{PA,blood}} = \sigma^2[\mathrm{PA}_{k,\mathrm{blood}}]$, one finally gets the following expression for the total variance image given as Eq. 3 in the manuscript:

$$\sigma^2_{\mathrm{PA}}(\lambda,\mathbf{r}) = [1+\sigma^2_{\mathrm{laser}}(\lambda)] \times \sigma^2_{\mathrm{PA,blood}}(\lambda,\mathbf{r}) + \sigma^2_{\mathrm{laser}}(\lambda) \times m^2_{\mathrm{PA}}(\lambda,\mathbf{r}) + \sigma_n^2 \tag{S3}$$

# 2 Singular-value-decomposition (SVD) filtering

## 2.1 Principles

Eq. S3 indicates that the photoacoustic fluctuation image contains at least two terms in addition to the term of interest. While it would in principle be possible to estimate those two terms from the knowledge of the laser fluctuation, the mean image and the electronic noise, SVD filtering allows to also filter out other parasitic terms that would not be taken into account in the above expression (such as slow tissue motion for instance). In addition, SVD filtering is also a method of choice for Doppler ultrasound[2]. It was therefore applied to both photoacoustic fluctuation images and ultrasound images as the first processing step after beamforming. The SVD decomposition of a series of $N_{\mathrm{PA}}$ volumetric images is given by the following expression:

$$\mathrm{PA}_k(\mathbf{r}) = \sum_{i=1}^{N_{\mathrm{PA}}} \Lambda_i U_i(\mathbf{r}) V_i^*(k) \tag{S4}$$

In the decomposition above, $\{U_i(\mathbf{r})\}_{i=[1\ldots N_{\mathrm{PA}}]}$ and $\{V_i(k)\}_{i=[1\ldots N_{\mathrm{PA}}]}$ form orthonormal basis made of volumetric images and temporal series respectively, and $\{\Lambda_i\}_{i=[1\ldots N_{\mathrm{PA}}]}$ are the corresponding singular values. We note that the above expression requires that the number $N_{\mathrm{PA}}$ of volumetric photoacoustic image is smaller than the number of points $N_{\mathbf{r}}$ in each image, which was always the case in this work ($N_{\mathrm{PA}} \sim 10^3$, $N_{\mathbf{r}} \sim 10^5 - 10^6$). For the sake of clarity, we here illustrate SVD filtering in the case of photoacoustic imaging, but the expressions above are general ones. The SVD-filtered series of $N_{\mathrm{PA}}$ volumetric images are defined by

$$\mathrm{PA}_{k,\mathrm{SVD}}(\mathbf{r}) = \sum_{i=a}^{N_{\mathrm{PA}}} \Lambda_i U_i(\mathbf{r}) V_i^*(k) \tag{S5}$$

where the singular values are in descending order. Filtering out low-index singular values ($i < a$) aims at removing fluctuations $\delta_k$ from the laser. This is possible thanks to the very different spatio-temporal signatures between spatially incoherent fluctuations from flowing red blood cells on one hand and fluence induced fluctuations that are the same for all pixels of a given image on the other hand. The same principle applies for ultrasound Doppler, where SVD mostly aims at separating global tissue motion (spatially coherent) from blood flow (spatially incoherent).

### 2.2 Noise compensation for multispectral PAFI

For photoacoustic images, under the assumption that the SVD operation perfectly separates the fluctuations induced by the laser from the fluctuations of interest, the variance computed from the SVD-filtered stack of images is given by

$$\sigma^2_{\mathrm{PA,SVD}}(\lambda,\mathbf{r}) = \sigma^2_{\mathrm{PA,blood}}(\lambda,\mathbf{r}) + \sigma_n^2 \tag{S6}$$

An unbiased quantitative estimation of $\sigma_{\mathrm{PA,blood}}(\lambda,\mathbf{r}) = \sqrt{\sigma^2_{\mathrm{PA,SVD}}(\lambda,\mathbf{r}) - \sigma_n^2}$ thus requires to know $\sigma_n^2$, as a crucial step towards quantitative $SO_2$ values. $\sigma_n$ is a characteristic of the electronic system and beamforming step, and can be estimated either directly from a stack of beamformed images containing only noise, or equivalently from the measured standard deviation of the real-valued RF signals $\sigma_{n,RF}$. When beamforming signals with $n_{\mathrm{elts}} = 256$ elements, under the assumption that noise traces are independent on each channels, one gets $\sigma_n^2 = 2 \times n_{\mathrm{elts}} \times \sigma^2_{n,RF}$, which was used in this work to compute $\sigma_n$ from prior measurements of $\sigma_{n,RF}$. Note that the factor 2 comes from the fact that we used *complex-valued* signals to reconstruct images, while $\sigma_{n,RF}$ is the standard deviation of *real-valued* signals.

### 2.3 Influence of the lower bound $a$

Filtering out singular vectors with index below the lower bound $a$ is needed to eliminate the contribution of the conventional mean image to the fluctuation image, to reduce the background, and reveals vessels otherwise invisible because of the limited view detection. With the same embryo as that used for Fig. 6 of the main manuscript, Fig. S1 illustrates the influence of $a$ on the reconstructed fluctuation image and $SO_2$ values derived on two vessels with different oxygenation states (same vessels as in Fig. 6). Without SVD filtering (Fig. S1, $a = 1$), the mean (i.e conventional) image (second term of Eq. 3) is dominant in the total fluctuation images, with a significant background caused by the probe sparsity. If $a$ is too high ($a \geq 50$ on Fig. S1), the fluctuations of interest from blood flow also partially filtered out by the SVD filtering step, reducing the apparent fluctuation amplitude and the corresponding contrast to noise ratio. For a large range of values, typically $20 < a < 50$, the fluctuation images are qualitatively similar. As also illustrated in Fig. S1, the $SO_2$ values may depend on $a$ (red curve) or may be quite insensitive to $a$ (blue curve). The next section explains the method that was designed to provide a user-independent choice of $a$, based on a contrast-to-noise criterium.

### 2.4 Automated determination of the lower bound $a$

We used the approach summarized in Fig.S2 to determine $a$ in a user-independent way from the optimization of a contrast-to-noise (CNR) metrics. We define the contrast-to-noise (CNR) of an object of interest on a fluctuation image from the following principle:

$$\mathrm{CNR(object)} = \frac{\mathrm{fluctuation[object] - background}}{\mathrm{spatial\ fluctuation\ of\ the\ background}} \tag{S7}$$

In the expression above, fluctuation[object] is defined as the variance signal $\sigma_{\mathrm{PA}}(\lambda,\mathbf{r})$ spatially averaged over the object, while the background is estimated from the same quantity spatially averaged everywhere else. The denominator is estimated through the spatial standard deviation of the background. Estimating the average variance fluctuation of the object requires not only that the position of the object may be first defined, but also that the fluctuation may be estimated over the object with no bias from parasitic source of fluctuations. In all our experiments, the pulse energy fluctuations induced a significant contribution of the second term in Eq.S3 (mean image) to the total fluctuation. Optimizing the CNR over the whole object would lead to optimize this parasitic fluctuation term, whereas the objective of the SVD step is to filter out this contribution. We thus estimated fluctuation[object] only for the part of the object that did not contribute to the mean image $m_{\mathrm{PA}}(\lambda,\mathbf{r})$. This approach requires that two binary masks $B_{m_{\mathrm{PA}}}(\mathbf{r})$ and $B_{\sigma_{\mathrm{PA}}}(\mathbf{r})$ are defined from the conventional and fluctuation images respectively, in order to compute a relevant CNR value. Once these masks are obtained, a global CNR value averaged over all wavelengths was defined explicitly and computed as

$$\mathrm{CNR}(a) = \frac{1}{M_\lambda}\sum_{j=1}^{M_\lambda} \frac{\left\langle B_{\sigma_{\mathrm{PA}}}(\mathbf{r}) \times (1 - B_{m_{\mathrm{PA}}}(\mathbf{r})) \times \sigma^2_{\mathrm{PA}}(\lambda_j,\mathbf{r})\right\rangle_{\mathbf{r}} - \left\langle (1 - B_{\sigma_{\mathrm{PA}}}(\mathbf{r})) \times \sigma^2_{\mathrm{PA}}(\lambda_j,\mathbf{r})\right\rangle_{\mathbf{r}}}{\mathrm{std}[(1 - B_{\sigma_{\mathrm{PA}}}(\mathbf{r})) \times \sigma^2_{\mathrm{PA}}(\lambda_j,\mathbf{r})]} \tag{S8}$$

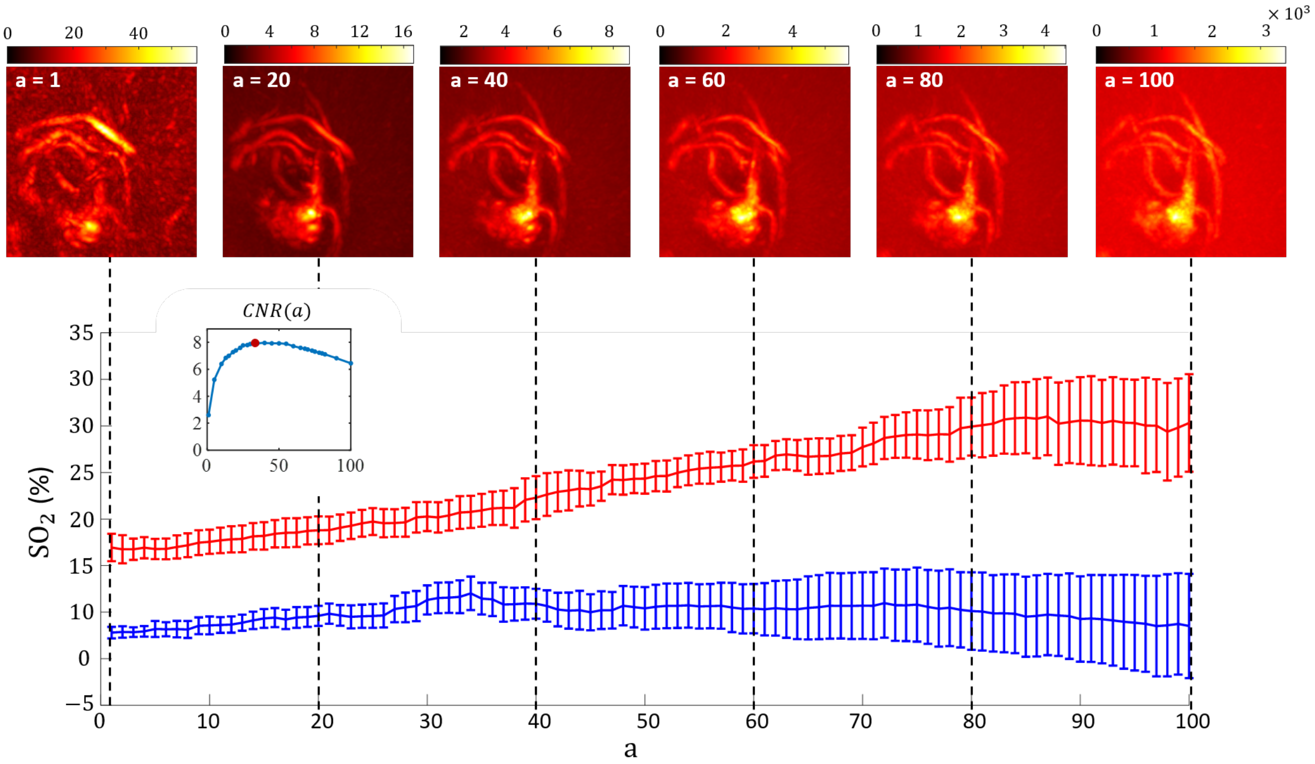



**Figure S1.** Influence of the lower bound $a$ on photoacoustic fluctuation images. The sample and data are the same as for Fig. 6 of the main manuscript. The fluctuations images shown as a function of $a$ are obtained from measurement at a single optical wavelength ($\lambda$=720 nm). The red and blue curves correspond to $SO_2$ values measured in the same two vessels as for Fig. 6. The final values were obtained for $a = 33$, determined from maximizing a contrast to noise ratio (CNR) as described in the next section.

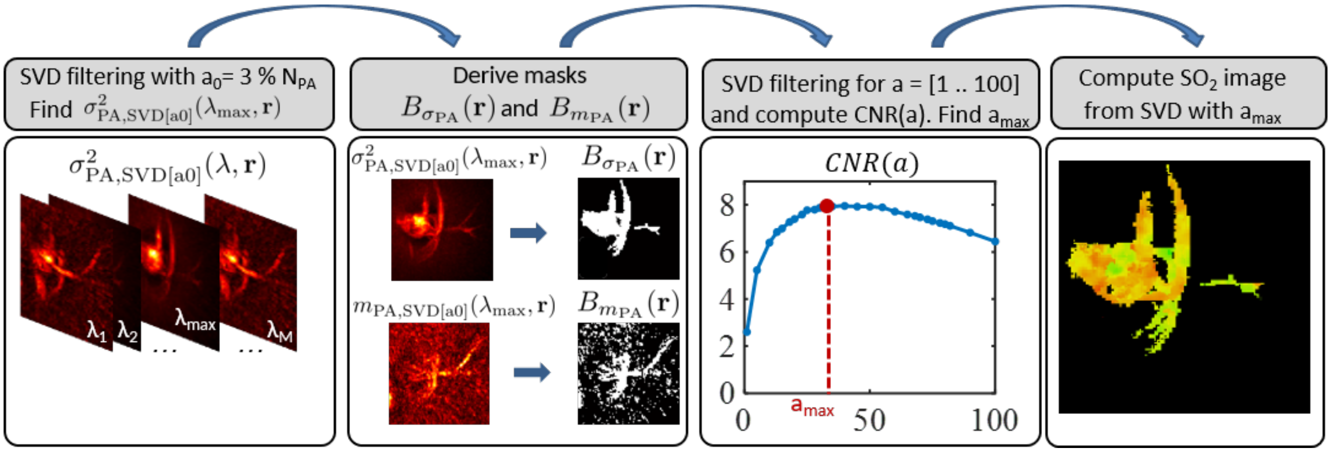



**Figure S2.** Illustration of the deterministic and user-independent method used to define a lower bound $a_{\text{max}}$ from a contrast-to-noise (CNR) optimization. A preliminary SVD filtering step with a fixed lower bound $a_0$ is used to define binary masks over the object of interest. SVD filters are then applied for different values of $a$ and a CNR metrics based on the masks is computed for each value of $a$. $SO_2$ computations are performed from the SVD filter with lower bound $a_{\text{max}}$.

The first step of the proposed method, as described in Fig.S2, is to define the two binary masks $B_{\sigma_{\text{PA}}}(\mathbf{r})$ and $B_{m_{\text{PA}}}(\mathbf{r})$. This was done by performing a preliminary SVD filtering with a fixed lower bound $a_0 = 3\% \times N_{\text{PA}}$, which systematically provided fluctuations images of sufficient quality to define masks for the fluctuating object of interest. From the resulting stack of $M_\lambda$ fluctuation images, we identified the wavelength $\lambda_{\max}$ corresponding to the fluctuation image with the highest peak value. The binary masks were then finally defined as :

$$B_{\sigma_{\text{PA}}}(\mathbf{r}) = \sigma^2_{\text{PA,SVD[a0]}}(\lambda_{\max}, \mathbf{r}) > [\sigma_n^2 + 3.5 \ \times \text{std}\left[\sigma^2_{\text{PA,SVD[a0]}}(\lambda_{\max}, \mathbf{r})\right]] \tag{S9a}$$

$$B_{m_{\text{PA}}}(\mathbf{r}) = m_{\text{PA,SVD[a0]}}(\lambda_{\max}, \mathbf{r}) > [< m_{\text{PA,SVD[a0]}}(\lambda_{\max}, \mathbf{r}) >_{\mathbf{r}} + 3.5 \ \times \text{std}\left[m_{\text{PA,SVD[a0]}}(\lambda_{\max}, \mathbf{r})\right]] \tag{S9b}$$

Importantly, the resulting masks are quite insensitive to the constant factors (3 % in the definition of $a_0$ and the factor 3.5 in weighting the standard deviations above) that were chosen heuristically. SVD filtering was then computed for all possible values of a in the range [1-100], along with the corresponding CNR. Finally, the final value $a_{\max}$ chosen for the final SVD filtering step is the one that maximize CNR(a), as illustrated in Fig. S2.
For all the experiments on embryos, the method proposed above successfully yielded values of $a$ that fell in the range for which $SO_2$ values depend little on $a$ (see Fig. S1 for instance), a necessary condition for meaningful quantitative imaging. We note however that the proposed method is one method out of many possible alternatives. Here, we only focused on finding some relevant value for $a$ in a deterministic and user-independent way, and it was out of our scope to optimize the method to choose $a$.

# 3 Ultrasound Doppler

## 3.1 Data processing

We briefly summarize the processing steps involved in the reconstruction of the various Doppler modes involved in our work, and provide the relevant references in the literature. For all Doppler modes, as for photoacoustic imaging, a prior SVD-filtering step was applied to the stack of volumetric ultrasound images $\text{US}_k(\mathbf{r})$, leading to a stack of volumetric ultrasound images $\text{US}_{k,\text{SVD}}(\mathbf{r})$.

**Power Doppler (US-PwD).** US-PwD volumetric images were reconstructed following the approach introduced by Demene *et al.*[2], by simply computing the fluctuation image defined here as:

$$\sigma_{\text{US}}(\mathbf{r}) = \sqrt{\text{var}[\text{US}_{k,\text{SVD}}(\mathbf{r})]} \tag{S10}$$

**Directional Power Doppler (US-dPwD).** A Fourier transform was applied pixelwise along the temporal dimension $k$ of the stack of SVD-filtered images $\text{US}_{k,\text{SVD}}(\mathbf{r})$. The resulting spectrum was then split into positive and negative frequencies $\widetilde{\text{US}}^+_{\text{SVD}}(f, \mathbf{r}) = \widetilde{\text{US}}_{\text{SVD}}(f > 0, \mathbf{r})$ and $\widetilde{\text{US}}^-_{\text{SVD}}(f, \mathbf{r}) = \widetilde{\text{US}}_{\text{SVD}}(f < 0, \mathbf{r})$, which can be transformed back into the temporal domain to yield $\text{US}^+_{k,\text{SVD}}(\mathbf{r})$ and $\text{US}^-_{k,\text{SVD}}(\mathbf{r})$. The two directional Power Doppler images are then defined as:

$$\sigma^+_{\text{US}}(\mathbf{r}) = \sqrt{\text{var}[\text{US}^+_{k,\text{SVD}}(\mathbf{r})]} \tag{S11a}$$

$$\sigma^-_{\text{US}}(\mathbf{r}) = \sqrt{\text{var}[\text{US}^-_{k,\text{SVD}}(\mathbf{r})]} \tag{S11b}$$

Note that $\sigma^+_{\text{US}}(\mathbf{r})$ and $\sigma^-_{\text{US}}(\mathbf{r})$ can also be conveniently (and strictly equivalently) computed directly in the Fourier domain by integrating the corresponding power spectra $|\widetilde{\text{US}}^+_{\text{SVD}}(f, \mathbf{r})|^2$ and $|\widetilde{\text{US}}^-_{\text{SVD}}(f, \mathbf{r})|^2$.

**Color Doppler (US-coD).** The ultrasound color Doppler volumetric image were defined as $v_{z,\text{US}}(\mathbf{r}) = \frac{c_{\text{US}}}{2f_c} \times f_D(\mathbf{r})$, where the Doppler frequency shift $f_D$ is estimated from the first moment of the Power Doppler spectrum[3]:

$$f_D(\mathbf{r}) = \frac{\int f |\widetilde{\text{US}}_{\text{SVD}}(f, \mathbf{r})|^2 \text{d}f}{\int |\widetilde{\text{US}}_{\text{SVD}}(f, \mathbf{r})|^2 \text{d}f} \tag{S12}$$

**Pulsed Doppler.** Pulsed (i.e. time-resolved) Doppler values $v_{z,\text{US}}(\mathbf{r}, t_k)$ may be computed at any location $\mathbf{r}$ from the same formula as for color Doppler, by replacing the full spectrum $\widetilde{\text{US}}_{\text{SVD}}(f, \mathbf{r})$ by spectrograms $\widetilde{\text{US}}_{\text{SVD}}(f, \mathbf{r}, t_k)$. In the example presented in the next section, each spectrogram was computed over a temporal window with a temporal width of 18 time samples, corresponding to a temporal resolution of $\Delta T = \frac{18}{200 \text{ Hz}} = 90$ ms, sufficient to follow cardiac cycles.

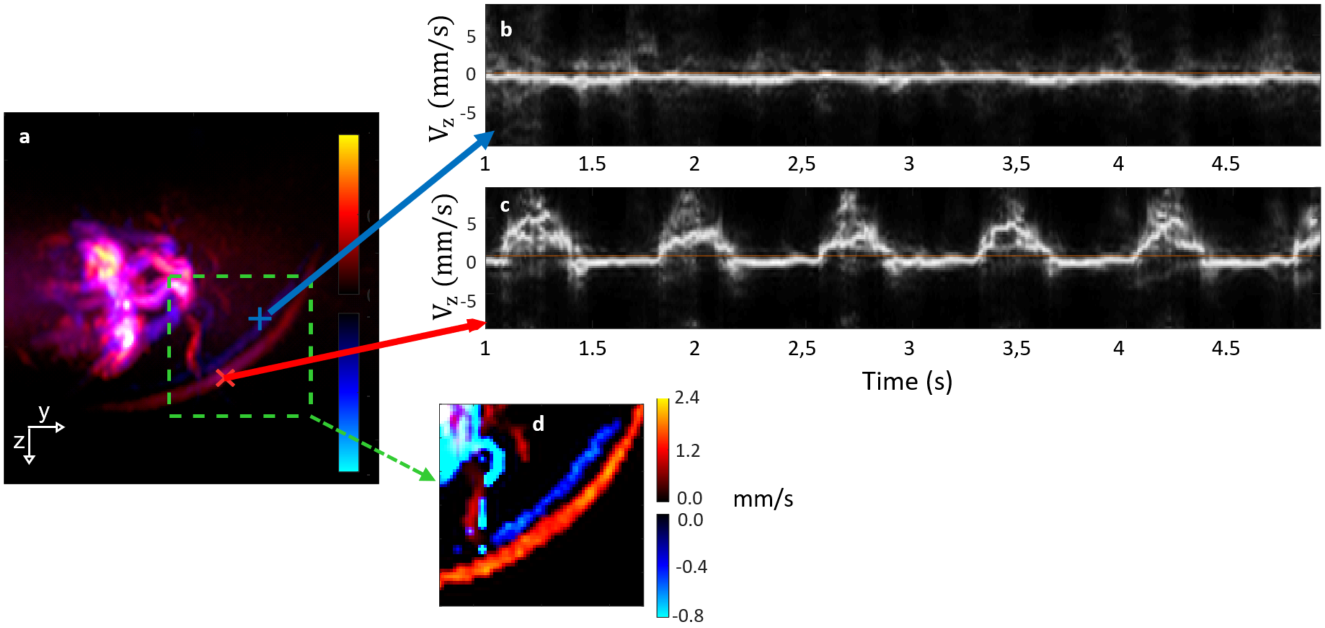


**Figure S3.** (a) Directional Power Doppler image. (d) Color Doppler image from the dashed region of interest. Pulsed Doppler values computed respectively at one pixel in the blue vessel (b) and in the the red vessel (c). The pulsed Doppler information allows identifying the red vessel as an artery and the blue vessel as a vein.

### 3.2 An example of pulsed Doppler analysis

Figure S3 illustrates the capability of our method to provide a pulsed Doppler analysis from the volumetric ultrasound image. The directional Power Doppler and color Doppler images shown on Figure S3(a) and (d) allow identifying two distinct vessels with opposite directions in the dashed region of interest. Additional complementary information can then be obtained through the pulsed Doppler analysis carried out at a given pixel on each vessel, as shown in Figure S3(b) and (c). While the Power Doppler and Color Doppler can in general not identify arteries from veins without further information on the vessels, the temporal traces of the pulsed Doppler signals permit such identification: the more pulsatile flow with higher peak velocities observed on the red-colored vessel is characteristic of an artery.

## 4 Tables of parameters

| | Photoacoustics | | | | Ultrasound |
|---|---|---|---|---|---|
| | $M$ | $M_{SO_2}$ | $N_{\text{PA}}$ | $a$ | $N_{\text{US}}$ |
| Figure 2 (#1) | 10 | – | 1000 | 18 | 1000 |
| Figure 2 (#2) | 10 | – | 3000 | 33 | 1000 |
| Figure 3 | 10 | 10 | 5000 | 70 | – |
| Figure 4 | 10 | 7 | 1000 | 33 | – |
| Figure 5 | – | – | – | – | 1000 |
| Figure 6 | 10 | 10 | 3000 | 33 | 1000 |
| Figure S1 | 10 | 10 | 3000 | var. | – |
| Figure S3 | 10 | – | – | – | 1000 |

**Table S1.** Acquisition and processing parameters for all fluctuation images. $M$: total number of acquired wavelengths. $M_{SO_2}$: number of wavelengths used for $SO_2$ estimation. $N_{\text{PA}}$ and $N_{\text{US}}$: number of images in the SVD-filtered stacks. $a$: lower bound for the SVD filtering step.

| $\lambda_j$ (nm) | 700 | 720 | 740 | 760 | 780 | 800 | 820 | 840 | 860 | 880 |
|---|---|---|---|---|---|---|---|---|---|---|
| $\mu_{\text{Hb}}$(cm$^{-1}$.M$^{-1}$) | 1794 | 1325 | 1115 | 1548 | 1075 | 761 | 693 | 692 | 694 | 726 |
| $\mu_{\text{HbO}_2}$(cm$^{-1}$.M$^{-1}$) | 290 | 348 | 446 | 586 | 710 | 816 | 916 | 1022 | 1092 | 1154 |

**Table S2.** Model absorption values for oxy- and deoxyhaemoglobin used to derive $SO_2$ oxygenation values from fitting the data. Values taken from the OMLC reference website[4].

## References


1. Vilov, S., Godefroy, G., Arnal, B. & Bossy, E. Photoacoustic fluctuation imaging: theory and application to blood flow imaging. *Optica* **7**, 1495–1505 (2020).

2. Demené, C. *et al.* Spatiotemporal clutter filtering of ultrafast ultrasound data highly increases doppler and fultrasound sensitivity. *IEEE transactions on medical imaging* **34**, 2271–2285 (2015).

3. Osmanski, B.-F., Maresca, D., Messas, E., Tanter, M. & Pernot, M. Transthoracic ultrafast doppler imaging of human left ventricular hemodynamic function. *IEEE transactions on ultrasonics, ferroelectrics, frequency control* **61**, 1268–1275 (2014).

4. Prahl, S. Optical absorption of hemoglobin. https://omlc.org (1999).
